# Supplementary material for: Lessons from the evaluation of the South African National Female Condom Programme
Source: PLoS One. 2020 Aug 13;15(8):e0236984. doi: 10.1371/journal.pone.0236984 (PMC7425948; doi:10.1371/journal.pone.0236984)
Supplement: S1 File — (PDF) [file pone.0236984.s001.pdf]

Evaluation of the National South African Female Condom Programme: Investigating Factors Associated with Uptake and Sustained Use

Key informant discussion guide: Policy Maker

PARTICIPANT ID NUMBER:	DATE (DD/MMM/YY):	START TIME:	END TIME:	INTERVIEWER INITIALS:

[Read to participant]

MatCH Research is conducting a national female condom (FC) evaluation which aims to identify factors associated with uptake and continued use of FCs among couples. Today I would like to ask you some questions about your organization and its role in policy relating to condom programming. The questions will focus on different aspects of policy-making including distribution, procurement, training, advocacy and promotion of male and female condoms, barriers to male and female condom use, target groups and coordination of the condom programme with other in-country and external organizations.

**A. BACKGROUND**<br>***Please complete information in spaces below:***		
1.	Organization name:	
2.	Organization type:	
3.	Participant job title:	
4.	Office/Department:	
5.	Number of years in current position:	

[Turn on digital recorder now]

**B. YOUR ORGANISATION AND ITS ROLE IN POLICY-MAKING FOR THE CONDOM PROGRAMME**

This section explores the aims and objectives of your organization in relation to policy development for the condom programme		
	**Questions**	**Probes**
6.	Can you briefly describe your organization?	a. DoH/local /international /affiliate of international organization?<br>b. For profit or not-for-profit?<br>c. How is the organization funded?
7.	What is your organization's role in policy and/or planning for condom programming?	a. Is it advisory/policy development/supply systems management/monitoring and evaluation/other?

**C. BACKGROUND TO NATIONAL COMPREHENSIVE CONDOM PROGRAMMING**<br><br>*This section will ask some general questions about the structure and history of the condom programme.*		
8.	How is management of national condom programming organized or structured?	a. Is it seen as one national condom programme or is it decentralized to provinces?<br>b. Who is primarily responsible for coordination?<br>c. Are male and female condom programmes integrated or separate?<br>d. How often are national-level meetings regarding implementation held?
9.	Please give a brief overview of the history of the condom programme?	a. Over the last 5-10 years, has the programme increased in size, stayed the same or contracted?
10.	Which services/programmes are involved in condom promotion and distribution? (FP, STI, HIV, ART, HCT, PMTCT, ANC/PNC, MMC, PHC)	a. Which of these services are integrated?<br>b. Is condom programming integrated across these services?

**D. POLICY IN COMPREHENSIVE CONDOM PROGRAMMING**<br><br>*This section explores policy-making relating to all aspects of condom programming including coordination, demand creation, distribution and supply, and support.*		
11.	Who is primarily responsible for developing national policy for condom programming?	a. Is it only DoH? Others involved?
12.	What is the process of policy development regarding condom programming?	a. How often do policy development meetings take place?<br>b. How are other partners involved?<br>c. Through which channels does each contribute?<br>d. Is any external technical assistance available for policy development? (Local/national/international agencies)
13.	To what extent is policy-making for condom programming (male and female) decentralized?	a. Do individual provinces/ develop their own policies?<br>b. Are provinces represented at a national level?<br>c. How do national policy directives interact with local policy?
14.	Is there a comprehensive and integrated national strategy for male and female condoms?	a. Current condom policy progress?<br>b. Please give details regarding:<br>i. Leadership and coordination;<br>ii. Demand, access and utilization,

		iii. Supply and commodity security,<br>iv. Support (advocacy, research, capacity and institutional strengthening, M&E, documentation, dissemination)
15.	Is there a multi-year operational plan and budget for male and female condom programming?	a. How many years of implementation does it cover?<br>b. What does it cover?<br>c. Is it linked to a national commodity security plan?<br>d. Is it linked to the existing logistics system for essential drugs and HIV-related commodities?
16.	What other key national policy documents relate to male and female condom programming?	a. Please describe the significance of each. Probe for policies in areas of:<br>i. Health (SRH, FP, ANC, PMTCT, HIV/STI/TB, HCT, MMC)<br>ii. Education (primary, secondary and tertiary)<br>iii. Workplace<br>b. Are male and female condoms included on the national essential drugs list or equivalent?
17.	According to national policy, where should male and female condoms be obtainable by different population groups?	a. CSWs, pregnant women, men and youth (probe for each group).<br>b. Which public sector programmes? (Probe for health - SRH/HIV/STI/FP/ANC/PMTCT/MMC, education, workplace, prisons, etc.)<br>c. Private sector?<br>d. Social marketing sector?<br>e. NGO sector?
18.	At present condoms are free in the public sector and are donated to NGOs. Will this free condom policy to all organisations remain in place in the future?	a. If demand increases? Is this sustainable? Will it be different for male and female condoms
19.	Which specific groups are prioritized in condom programming policy? Please describe priority actions for each group.	a. CSWs, pregnant women, men and youth (probe for each group).<br>b. Please describe policies on schools-based and youth friendly health services relating to condom promotion and distribution.<br>c. Is there a specific policy focusing on one or more vulnerable groups and their SRH needs?
20.	How do policy-makers communicate with implementers of the condom programme?	a. Which main programmes/organizations are involved in implementation?<br>b. How is policy disseminated? To what level?<br>c. What partnerships or channels of communication/support exist between policy-makers and programme managers?
21.	Please tell me more about the role of condom social marketing (CSM) in the national condom programme?	a. What partnerships exist with policy-makers?<br>b. How do policy-makers ensure that CSM activities are coordinated/aligned with national priorities?<br>c. Which regulatory processes govern CSM?

**E. FINANCIAL RESOURCES**<br><br>*This section explores financing for comprehensive condom programming, regarding current mechanisms, sustainability and future plans.*		
22.	How are male and female condoms financed?	a. <u>National</u>:<br>i. Do departments/provinces have individual budgets or national budget only?<br>b. <u>If any donor funded</u>:

		i. What mechanisms are used? (Direct commodity supply/Budgetary support/Project or programme support/other)<br>ii. What level of country commitment exists to achieve self-sufficiency in funding male and female condoms?<br>c. Is current funding sufficient to meet demand?<br>d. For how long is current funding guaranteed?<br>e. Are there any other problems with the current funding mechanism?
23.	Are there any funding gaps in the operational plan?	a. Where do these exist?<br>b. Have gaps ever caused condom stock-outs in the last 5 years?<br>c. What is being done to fill these gaps?<br>d. What else does government do to advocate for secure funds for implementation of the condom strategy and operational plan?

**F. HUMAN RESOURCES AND INSTITUTIONAL CAPAPCITY**<br><br>*This section explores your knowledge and experience of human resources and institutional capacity to effectively implement the condom programme from supply chain management to provider distribution and promotion.*		
24.	How is forecasting undertaken for male and female condom demand and supply?	a. What methods/systems are used?<br>b. At what level does forecasting occur? (National only, or also provincial and local)<br>c. Who is responsible for forecasting?
25.	How are male and female condoms procured?	a. What protocols and processes exist for public sector procurement?<br>b. Who is responsible for this?<br>c. What current tenders/contracts exist?<br>d. Does procurement occur only at a national level, or may individual departments procure/receive condoms by other means?<br>e. Must NGO/CSM/private sector organizations access publically-procured condoms or do parallel procurement systems exist?<br>f. Are public-sector condoms re-packaged/re-branded in country?
26.	What is the national availability of M&F condoms?	a. How many male and female condoms are currently available in country per male/female of reproductive age per year?<br>b. Does this match current levels of demand?<br>c. How has this changed over the past 5 years?
27.	Please describe the distribution chain for male and female condoms.	a. What national and regional warehousing facilities exist? What is their storage capacity?<br>b. How are condoms transported to and from these warehouses? What infrastructure facilitates this?<br>c. Is supply chain management undertaken within the public sector or outsourced? How is this monitored?<br>d. What guidelines or protocols govern supply chain management?<br>e. How do primary and secondary distribution sites obtain condoms? At what cost? Is it a push or pull system?<br>f. How do CSM, NGO and private sector organizations obtain condoms from the public sector?<br>g. Do stock-outs occur? At what level?
28.	What capacity building support is available for procurement and logistics personnel?	a. Employment of logistics officers and managers?<br>b. Technical assistance?<br>c. In-service training?<br>d. Training of trainers?<br>e. What training materials are available? (Manuals, guidelines, protocols)

		f. Supervision or support channels?
29.	What capacity building support is available for programme managers and service providers?	a. In-service training?<br>b. Training of trainers?<br>c. What training materials have been developed or adapted (Manuals, guidelines, demonstration models)<br>d. Supervision or support channels?
30.	What are the major human resource and institutional strengths and gaps relating to the condom supply chain?	a. Where do gaps exist?<br>b. How can these be utilized/filled?<br>c. What has already been done to strengthen human resources and institutional capacity?

**G. CREATING AND SUSTAINING DEMAND FOR CONDOMS**<br><br>*This section explores the actions taken at a policy level to mobilize communities and increase acceptability and demand for both male and female condoms, to ensure correct and consistent use by those at risk.*		
31.	What research has been or is being conducted to inform policy-making for male and female condom programming?	a. Does this cover market research, target audience segmentation, knowledge, attitudes and practices?<br>b. Does this prioritize any vulnerable groups? (CSWs, pregnant women, men and youth)
32.	Has a communication strategy to promote correct and consistent use of condoms been implemented?	a. Please describe<br>b. What are the key messages, target audiences and communication channels?<br>c. Are these the same for male and female condoms?<br>d. Who is responsible for implementation?<br>e. What implementation partnerships have been established?<br>f. How is this coordinated at a national/provincial/local level?
33.	What creative or non-traditional outlets are employed for condom distribution or promotion?	a. For example dispensers, salons, youth centers.<br>b. How do these target vulnerable groups? (CSWs, pregnant women, men and youth)<br>c. Are these the same for male and female condoms
34.	What else is being done to mobilize communities to ensure a supportive environment for male and female condom use?	
35.	In your opinion, what are the main barriers and facilitators of a successful condom programme (I.e. male and female condoms being accessible and acceptable to all those who need them, and therefore used correctly and consistently)	a. Political, economic, infrastructure/capacity, religious, social and cultural factors.<br>b. To what extent are male or female condoms stigmatized, for example by being associated with promiscuity or high risk groups?<br>c. How can these barriers be overcome?<br>d. How is this accounted for in policy?
36.	Female condoms are more expensive than male condoms. Are there any concerns about migration of	

	users from MCs to FCs but no increase in overall protection?	

**H. STRENGTHENING ADVOCACY AND ENGAGING THE MEDIA**<br><br>*This section explores actions taken at a policy level to strengthen advocacy through civil society, media and other partners, for widespread accessibility and correct and consistent use of male and female condoms.*		
37.	How are policy and regulatory issues regarding access to and use of male and female condoms identified and dialogue established?	a. Stakeholder meetings?<br>b. Other channels?
38.	How do national and provincial governments interact with media regarding condom programming?	a. What media outreach is undertaken?<br>b. Is there a communication strategy to engage the media?<br>c. Are sensitization and skills-building workshops available for journalists and members of the mass media?
39.	What other channels exist for building advocacy?	a. National condom champions?<br>b. Coalitions and partnerships with civil society and other segments of society?<br>c. How are these nurtured? (Mission statement, roles and responsibilities, advocacy plan)

**I. MONITORING AND EVALUATION**<br><br>*This section asks questions about methods and functionality of the monitoring and evaluation system used to assess overall performance of the condom programme.*		
40.	What systems are used to monitor and evaluate the condom programme?	a. Please describe the reporting system for public sector condom supply and distribution.<br>b. Is this integrated with other information systems?<br>c. What targets are set for procurement and distribution?<br>d. What other indicators are used in terms of overall performance of the programme? How is data on these gathered?<br>e. Are any specific data gathered on high risk groups? (CSWs, pregnant women, men and youth)<br>f. How do CSM, NGO and private sector organizations report on their condom programming activities?
41.	What institutional and HR capacity exists for M&E?	a. Who is responsible for data collection/analysis?<br>b. What training and support is available for M&E personnel?
42.	How is M&E information shared and utilized?	a. How often are reports collated?<br>b. How often are targets and indicators reviewed and updated?<br>c. How often are programme evaluations undertaken, including programme impact?

**J. THE FEMALE CONDOM PROGRAMME**<br><br>*This section asks specific questions relating to female condom programming. We would like to know how this differs from male condom programming and what special considerations are required. Some of this information may have already been covered in previous sections, so we will try our best to avoid repetition.*

43.	There are several brands of female condom available internationally. Which brands are available in South Africa?	a. Currently procurement for the public sector?<br>b. Why these brands? (Probe for cost, regulatory issues, tendering, QA etc.)<br>c. This changed since initial rollout? If so, why?<br>d. Are any other brands available in the CSM/NGO/private sector?<br>e. Are there plans to introduce new brands to any sector?<br>f. How will programming several brands work?
44.	How is technical expertise and advocacy for female condom programming ensured at a national policy level?	a. FC working group or technical committee?<br>b. What are the roles/responsibilities of such a group or individual?
45.	From a policy point of view, what are the main differences between female and male condom programming?	a. If necessary probe for acceptability, supply issues, cost, etc.
46.	What are the strengths and weaknesses of the female condom programme?	a. Relating to access, coverage, uptake etc.<br>b. What action is being taken to address these?
47.	What plans exist to scale up female condom programming?	a. What priority actions have been identified?<br>b. What is currently being done to achieve this?

**K. CONCLUSION**		
48.	Do you have anything else you would like to add about anything we have talked about today?	

This concludes our interview today. Thank you very much for your time.
