# Supplementary material for: Lessons from the evaluation of the South African National Female Condom Programme
Source: PLoS One. 2020 Aug 13;15(8):e0236984. doi: 10.1371/journal.pone.0236984 (PMC7425948; doi:10.1371/journal.pone.0236984)
Supplement: S2 File — (PDF) [file pone.0236984.s002.pdf]

Evaluation of the National South African Female Condom Programme: Investigating Factors Associated with Uptake and Sustained Use

PROVIDER INTERVIEW

NOTE.

*FOR EACH OF THE QUESTIONS LISTED BELOW, MARK AN **X** IN THE BOX FOR THE OPTION THAT REPRESENTS THE PARTICIPANT'S RESPONSE OR WRITE PARTICIPANT'S OWN WORDS (VERBATIM) FOR OPEN-ENDED QUESTIONS.*

**Facility/site Name/Section**	
**Type of provider**	❑ 1= Registered nurse<br>❑ 2= Enrolled nurse<br>❑ 3= Enrolled assistant<br>❑ 4= Lay counselor<br>❑ 5= Medical doctor<br>❑ 6= Volunteer<br>❑ 77= Other<br>*SPECIFY*______________________________
**Interviewer code**	
**Date data collected (Date/month/year)**	______/____________/ ________
**Interview start time**	
**Interview stop time**	
**Results codes**	❑ 1= Complete<br>❑ 2= Partially complete<br>❑ 3= Refused to continue

CHECKED BY ……………………………………………………………………..

DATE CHECKED ……………………………………………………………...

**DATA ENTRY BY** …………………………………………………………………..
**DATA ENTRY DATE** ………………………………………………………………

**1. BACKGROUND INFORMATION**<br>I would like to start the interview by asking you some questions about yourself.		
**1.1**	**Please tell me how old are you?**	Completed years ______
**1.2**	**Gender**	❑ 1= Female<br>❑ 2= Male
**1.3**	**What is your highest academic qualification?**	❑ 1= Diploma<br>❑ 2= Degree<br>❑ 3= Certificate<br>❑ 77= Other,<br>*SPECIFY*___________________________
**1.4**	**How many years have you been working as a (job role given above), including your training?**	Number of years:_________________
**1.5**	**Which of the following services are you involved in providing at the moment (in last month)?**<br>*MARK ALL GIVEN ANSWERS*	❑ 1= PHC (non-specific)<br>❑ 2= Family planning<br>❑ 3= STI<br>❑ 4= ARV<br>❑ 5= HCT<br>❑ 6= SGBV<br>❑ 7= Counseling<br>❑ 8= Condom distribution<br>❑ 9= CTOP<br>❑ 10= Cervical cancer screening<br>❑ 77= Other,<br>*SPECIFY*_____________________

**2.0**	**TRAINING**
**2.1**	**Have you ever been trained in [read topic area]? Did this training take place in the last 2 years? Do you need more training in this area?**<br>READ ALOUD AND FOR EACH SERVICE.

	Topic area	Ever trained	Trained in last 2 years?	Need more training
**a**	**Family planning (counselling & method)**	❑ 1= Yes<br>❑ 2= No	❑ 1= Yes<br>❑ 2= No	❑ 1= Yes<br>❑ 2= No

	b	**Female condom counselling & demonstration**	❑ 1= Yes<br>❑ 2= No	❑ 1= Yes<br>❑ 2= No	❑ 1= Yes<br>❑ 2= No
	c	**Male condom counseling & demonstration**	❑ 1= Yes<br>❑ 2= No	❑ 1= Yes<br>❑ 2= No	❑ 1= Yes<br>❑ 2= No
	d	**HCT/HIV counselling**	❑ 1= Yes<br>❑ 2= No	❑ 1= Yes<br>❑ 2= No	❑ 1= Yes<br>❑ 2= No
	e	**HIV prevention and risk (condoms, risk factors)**	❑ 1= Yes<br>❑ 2= No	❑ 1= Yes<br>❑ 2= No	❑ 1= Yes<br>❑ 2= No
	f	**HIV management (ART and wellness)**	❑ 1= Yes<br>❑ 2= No	❑ 1= Yes<br>❑ 2= No	❑ 1= Yes<br>❑ 2= No
	g	**SGBV services**	❑ 1= Yes<br>❑ 2= No	❑ 1= Yes<br>❑ 2= No	❑ 1= Yes<br>❑ 2= No
	h	**STI screening, prevention and treatment**	❑ 1= Yes<br>❑ 2= No	❑ 1= Yes<br>❑ 2= No	❑ 1= Yes<br>❑ 2= No
	i	**Cervical cancer screening**	❑ 1= Yes<br>❑ 2= No	❑ 1= Yes<br>❑ 2= No	❑ 1= Yes<br>❑ 2= No
	j	**HPV**	❑ 1= Yes<br>❑ 2= No	❑ 1= Yes<br>❑ 2= No	❑ 1= Yes<br>❑ 2= No
	k	**Safer conception for HIV-positive women**	❑ 1= Yes<br>❑ 2= No	❑ 1= Yes<br>❑ 2= No	❑ 1= Yes<br>❑ 2= No
	l	**Sexual health**	❑ 1= Yes<br>❑ 2= No	❑ 1= Yes<br>❑ 2= No	❑ 1= Yes<br>❑ 2= No
	m	**Taking sexual history**	❑ 1= Yes<br>❑ 2= No	❑ 1= Yes<br>❑ 2= No	❑ 1= Yes<br>❑ 2= No
	n	**Values clarification**	❑ 1= Yes<br>❑ 2= No	❑ 1= Yes<br>❑ 2= No	❑ 1= Yes<br>❑ 2= No
	o	**Other, *SPECIFY***	________________	________________	__________

**3.0 SEXUAL HEALTH, AND RISK-REDUCTION COUNSELING**		
**3.1.**	**Do you ask for sexual history information from your clients? (e.g. whether client is sexually active, number of partners, different sexual practices, such as vaginal, oral and anal sex, etc.)**	❑ 1= Yes<br>❑ 2= No<br>❑ 3= Depends on client/consultation
**3.2**	**Are there any types of clients that you might feel uncomfortable taking a sexual history from or discussing their sexual behaviour?** *CHOOSE ALL THAT PROVIDER MENTIONS*	❑ 1= No group<br>❑ 2= Married women<br>❑ 3= Unmarried women<br>❑ 4= Married men<br>❑ 5= Unmarried men<br>❑ 6= Adolescent girls<br>❑ 7= Adolescent boys<br>❑ 8= Older women (45+)<br>❑ 9= Older men (45+)<br>❑ 10= Commercial sex workers

3.0 SEXUAL HEALTH, AND RISK-REDUCTION COUNSELING		
		❑ 11= MSM<br>❑ 77= Other, *SPECIFY*___________
3.3	**Which of the following sexual practices do you discuss when counseling clients on safe sex** *READ OUT EACH PRACTICE*	

	Practice	Practice discussed	Condom use discussed with practice
a.	**Oral sex**	❑ 1= Yes<br>❑ 2= No<br>❑ 3= Depends on client	❑ 1= Yes<br>❑ 2= No<br>❑ 3= Depends on client
b.	**Anal sex**	❑ 1= Yes<br>❑ 2= No<br>❑ 3= Depends on client	❑ 1= Yes<br>❑ 2= No<br>❑ 3= Depends on client
c.	**Vaginal practices**	❑ 1= Yes<br>❑ 2= No<br>❑ 3= Depends on client	❑ 1= Yes<br>❑ 2= No<br>❑ 3= Depends on client
d.	**Use of lubricant**	❑ 1= Yes<br>❑ 2= No<br>❑ 3= Depends on client	❑ 1= Yes<br>❑ 2= No<br>❑ 3= Depends on client
e.	**Other, *SPECIFY***	___________________<br>___________________	_____________________<br>_____________________

3.4	**When you counsel a patient who has an STI, what do you usually discuss?**<br>*CIRCLE ALL THAT PROVIDER MENTIONS. DO NOT READ OUT LIST.*	❑ 1= HIV/AIDS<br>❑ 2= HCT<br>❑ 3= Risk of having multiple partners<br>❑ 4= Abstinence during STI treatment<br>❑ 5= Partner referral for STI treatment<br>❑ 6= How to take treatment<br>❑ 7= Family planning<br>❑ 8= Condom use<br>❑ 9= SGBV<br>❑ 77= Other, *SPECIFY*_____________	

4.0 FEMALE AND MALE CONDOM COUNSELING AND DEMONSTRATION		
4.1	**In the last month, how often did you discuss male or female condom use with your clients in a one-to-one consultation?**	

Condom type	Frequency
**Female condom**	❑ 1= Never<br>❑ 2= Less than half the clients<br>❑ 3= Half the time<br>❑ 4= More than half<br>❑ 5= Almost all<br>❑ 6= Depended on client
**Male condom**	❑ 1= Never<br>❑ 2= Less than half the clients<br>❑ 3= Half the time

		❑ 4= More than half<br>❑ 5= Almost all<br>❑ 6= Depended on client	

4.2 **In the following list of services, with which type of client do <u>YOU routinely</u> raise the topic of condom use?**

	Service	Type of client	Which condom?		
a.	**PHC**	❑ 1= All clients<br>❑ 2= Female clients<br>❑ 3= Male clients<br>❑ 4= Young clients<18	❑ 1= Male<br>❑ 2= Female<br>❑ 3= Both		
b.	**FP**	❑ 1= All clients<br>❑ 2= Female clients<br>❑ 3= Male clients<br>❑ 4= Young clients<18	❑ 1= Male<br>❑ 2= Female<br>❑ 3= Both		
c.	**Pregnancy testing**	❑ 1= All clients<br>❑ 2= Female clients<br>❑ 3= Male clients<br>❑ 4= Young clients<18	❑ 1= Male<br>❑ 2= Female<br>❑ 3= Both		
d.	**TOP client**	❑ 1= All clients<br>❑ 2= Female clients<br>❑ 3= Male clients<br>❑ 4= Young clients<18	❑ 1= Male<br>❑ 2= Female<br>❑ 3= Both		
e.	**ANC**	❑ 1= All clients<br>❑ 2= Female clients<br>❑ 3= Male clients<br>❑ 4= Young clients<18	❑ 1= Male<br>❑ 2= Female<br>❑ 3= Both		
f.	**PNC**	❑ 1= All clients<br>❑ 2= Female clients<br>❑ 3= Male clients<br>❑ 4= Young clients<18	❑ 1= Male<br>❑ 2= Female<br>❑ 3= Both		
g.	**HCT**	❑ 1= All clients<br>❑ 2= Female clients<br>❑ 3= Male clients<br>❑ 4= Young clients<18	❑ 1= Male<br>❑ 2= Female<br>❑ 3= Both		
h.	**STI**	❑ 1= All clients<br>❑ 2= Female clients<br>❑ 3= Male clients<br>❑ 4= Young clients<18	❑ 1= Male<br>❑ 2= Female<br>❑ 3= Both		
i.	**ARV/HIV wellness**	❑ 1= All clients<br>❑ 2= Female clients<br>❑ 3= Male clients<br>❑ 4= Young clients<18	❑ 1= Male<br>❑ 2= Female<br>❑ 3= Both		
j.	**Cervical cancer screening**	❑ 1= All clients<br>❑ 2= Female clients	❑ 1= Male<br>❑ 2= Female		

			❑ 3= Male clients<br>❑ 4= Young clients<18	❑ 3= Both	
	**k.**	**GBV service/referral**	❑ 1= All clients<br>❑ 2= Female clients<br>❑ 3= Male clients<br>❑ 4= Young clients<18	❑ 1= Male<br>❑ 2= Female<br>❑ 3= Both	
	**l.**	**MMC**	❑ 1= All clients<br>❑ 2= Female clients<br>❑ 3= Male clients<br>❑ 4= Young clients<18	❑ 1= Male<br>❑ 2= Female<br>❑ 3= Both	

**4.3**	**Who usually begins the discussion about female or male condoms in a one-to-one consultation?**	❑ 1= I do<br>❑ 2= Depends on the client<br>❑ 3= The client usually asks	

4.4 **How much time do you spend on average in counseling a new condom user?**

Time	Female condom	Male condom
Number (mins)	_______:_______	_______:_______

4.5 **In the last year, how often have you counseled/given female condoms to <u>men?</u>**

❑ 1= Never counseled/given FCs to men
❑ 2= Rarely counseled/given FCs to men (less than monthly)
❑ 3= Occasionally counseled/given FCs to men (at least once a month)
❑ 4= Often counseled/given female condoms to a man (most weeks)

4.6 **When you talk about condoms, what do you usually discuss with the client?**
READ OUT AND TICK ALL THAT APPLY

	Topic	Female condom	Male condom
a.	**How to use (shows client)**	❑	❑
b.	**Dual protection**	❑	❑
c.	**HCT**	❑	❑
d.	**STI and HIV risk**	❑	❑
e.	**Importance of correct and consistent use**	❑	❑
f.	**Negotiation of condom use**	❑	❑
g.	**Condom use during pregnancy**	❑	❑
h.	**Gender-based violence**	❑	❑
i.	**What to do if it breaks**	❑	❑
j.	**Emergency contraception**	❑	❑
k.	**Storage**	❑	❑
l.	**Expiry date**	❑	❑
m.	**Other, *SPECIFY***	______________	______________

<table>
<tr><td>4.7</td><td>When you explain “how to use” a condom to a <u>new condom user,</u> do you demonstrate use?
<table>
<tr><th>Condom type</th><th>Demonstrate use?</th></tr>
<tr><td>Female condom</td><td>❑ 1= Yes, always
❑ 2= Yes, sometimes
❑ 3= No, unless asked by client
❑ 4= Never demonstrated</td></tr>
<tr><td>Male condom</td><td>❑ 1= Yes, always
❑ 2= Yes, sometimes
❑ 3= No, unless asked by client
❑ 4= Never demonstrated</td></tr>
</table></td><td>IF 1, GO TO Q4. 9 IF 2/3/4. GO TO Q4.8</td></tr>
<tr><td>4.8</td><td>If you do not always demonstrate (never or sometimes), why not?
<table>
<tr><th>Condom type</th><th>Reason</th></tr>
<tr><td>Female condom</td><td>❑ 1= No demonstration models.
❑ 2= No time in consultation.
❑ 3= Clients can read the instructions
❑ 77= Other,
SPECIFY______________________</td></tr>
<tr><td>Male condom</td><td>❑ 1= No demonstration models.
❑ 2= No time in consultation.
❑ 3= Clients can read the instructions
❑ 77= Other,
SPECIFY____________________________</td></tr>
</table></td><td></td></tr>
<tr><td>4.9</td><td>ASK THIS QUESTION FOR THOSE WHO SAID THEY ALWAYS OR SOMETIMES DEMONSTRATE IN Q4.6.
When you show a new user “how to use” a condom, how do you demonstrate use?
PROBE FOR MODEL- DILDO, PELVIC MODEL, OTHER
<table>
<tr><th>Condom type</th><th>Demonstration tool</th></tr>
<tr><td>Female condom</td><td>❑ 1= Demonstration model if available
Name type___________________
❑ 2= Using hand</td></tr>
<tr><td>Male condom</td><td>❑ 1= Demonstration model if available
Name type___________________
❑ 2= Using hand</td></tr>
</table></td><td></td></tr>
<tr><td>4.10</td><td>If a woman asks for advice on how to introduce a condom into her relationship, what do you usually tell her?

Female condom____

Male condom____</td><td></td></tr>
</table>

4.11	**If a woman returns to the facility with the following problems with use of the female condom, what do you suggest to her?**	

**Problem**	**Suggestions**
**a. User problem (insertion, uncomfortable)**	❑ 1= Counsel to try again<br>❑ 2= Suggest another method or stop using
**b. Partner does not want to use**	❑ 1= Counsel to try again<br>❑ 2= Suggest another method or stop using

**5.1**	**Are female condoms kept in the consultation rooms to give directly to clients?**	❑ 1= Yes, kept in all consulting rooms<br>❑ 2= Yes, kept in some consulting rooms<br>❑ 3= No, kept elsewhere
**5.2**	**Are male condoms kept in the consultation rooms to give directly to clients?**	❑ 1= Yes, kept in all consulting rooms<br>❑ 2= Yes, kept in some consulting rooms<br>❑ 3= No, kept in dispensers only

5.3 **How are clients informed about the availability of condoms in this facility/site?**

LIST ALL THE WAYS FOR EACH TYPE OF CONDOM IN TABLE BELOW

**Condom type**	**Channels**
**Female condom**	❑ 1= Leaflets/pamphlets in main waiting/reception area<br>❑ 2= Leaflets/pamphlets in consulting rooms<br>❑ 3= Posters/signs in main waiting/reception<br>❑ 1= Posters/signs in consulting rooms<br>❑ 1= The providers tell clients<br>❑ 1= Other *SPECIFY*__________________________
**Male condom**	❑ 1= Leaflets/pamphlets in main waiting/reception area<br>❑ 2= Leaflets/pamphlets in consulting rooms<br>❑ 3= Posters/signs in main waiting/reception<br>❑ 4= Posters/signs in consulting rooms<br>❑ 5= The providers tell clients<br>❑ 6= Other, *SPECIFY*__________________________

5.4 **How do you make condoms available to your clients?**

LIST ALL THE WAYS FOR EACH TYPE OF CONDOM IN TABLE BELOW

**Condom type**	**Channels**
**Female condom**	❑ 1= I give them to clients<br>❑ 2= Refer them to dispenser in facility<br>❑ 77= Other, *SPECIFY*_____________
**Male condom**	❑ 1= I give them to clients<br>❑ 2= Refer them to dispenser in facility<br>❑ 77= Other, *SPECIFY*_____________

5.5	**How many female condoms do you normally give to the following types of user?** *ASK FOR NUMBER AND RANGE*

Type of user	Number	Range	Suggested resupply interval
**New users**			❑ 1= Discussed with client<br>❑ 2= Suggested time ________(weeks)
**Repeat users**			❑ 1= Discussed with client<br>❑ 2= Suggested time ________(weeks)

5.6	**Thinking back over the last week (last 5 working days), have you personally given condoms to any clients?**

	Female Condom	Male Condom
**Female client**	❑ 1= Yes<br>❑ 2= No	❑ 1= Yes<br>❑ 2= No
**Male client**	❑ 1= Yes<br>❑ 2= No	❑ 1= Yes<br>❑ 2= No

6. FEMALE CONDOM KNOWLEDGE AND ATTITUDES

6.1 Please indicate whether you agree or disagree with the following statements. Please answer to the best of your ability.

		Agree	Disagree	Not Sure
a.	The female condom can be inserted several hours before vaginal sex.	❑	❑	❑
b.	Any type of lubricant can be used with the female condom.	❑	❑	❑
c.	Lubricant can be applied either inside or outside the female condom.	❑	❑	❑
d.	The female condom can be used during a woman's menstrual cycle.	❑	❑	❑
e.	The female condom is not recommended for use during anal sex.	❑	❑	❑
f.	The female condom should not be used more than once.	❑	❑	❑
g.	The material that the female condom is made of conducts heat better than latex.	❑	❑	❑
h.	The female condom should be twisted as it is removed.	❑	❑	❑
i.	Besides this facility, I know where clients can obtain female condoms.	❑	❑	❑

6.2	**If a client wants to use a condom and asks you which one to recommend, what would you suggest if you had them both available in your consulting room?**<br>❑ 1= I would suggest a female condom<br>❑ 2= I would suggest a male condom<br>❑ 3= I would suggest she has both available<br>❑ 4= It would depend on the client<br>❑ 77= Other, *SPECIFY*________________________________

**6.3**	**If a client asks if she should give up her hormonal method and just use condoms for pregnancy protection, what do you think about this?**<br>❑ 1= Yes, for either male or female condoms<br>❑ 2= Yes, for male condoms only<br>❑ 3= Yes, for female condom only<br>❑ 4= No, should use a back-up method (hormonals) for both MC and FC
**6.4**	**What contraceptive method/s would you recommend to an HIV-positive client?**<br>*IF SHE MENTIONS DUAL PROTECTION –HORMONAL METHOD PLUS CONDOM, ASK WHICH CONDOM AND INDICATE IN THE CONDOM COLUMNS. IF ONLY MENTIONS CONDOMS, ASK WHICH ONES. THIS QUESTION IS UNPROMPTED*

	Method	For hormonal methods, did they mention condoms?	Female condom	Male condom
**a.**	**Injectable**	❑ 1= Yes<br>❑ 2= No	❑ 1= Yes<br>❑ 2= No	❑ 1= Yes<br>❑ 2= No
**b.**	**Pills**	❑ 1= Yes<br>❑ 2= No	❑ 1= Yes<br>❑ 2= No	❑ 1= Yes<br>❑ 2= No
**c.**	**Implants**	❑ 1= Yes<br>❑ 2= No	❑ 1= Yes<br>❑ 2= No	❑ 1= Yes<br>❑ 2= No
**d.**	**IUD**	❑ 1= Yes<br>❑ 2= No	❑ 1= Yes<br>❑ 2= No	❑ 1= Yes<br>❑ 2= No
**e.**	**Sterilization**	❑ 1= Yes<br>❑ 2= No	❑ 1= Yes<br>❑ 2= No	❑ 1= Yes<br>❑ 2= No
**f.**	**Male condoms**	❑ 1= Yes<br>❑ 2= No		
**g.**	**Female condoms**	❑ 1= Yes<br>❑ 2= No		

**6.5**	**Some providers have used the female condom and others have never used a female condom. Have you ever used a female condom yourself with a current or previous partner?**	❑ 1= Yes<br>**IF YES, GO TO 6.7**<br>❑ 2= No<br>**IF NO, GO TO 6.6**
**6.6**	**IF NEVER used a female condom: What are some of the reasons why you have not tried to use a female condom?**<br>*MARK ALL THAT APPLY*<br>❑ 1= They are not available here/out-of-stock<br>❑ 2= Partner will object<br>❑ 3= Use other contraceptive<br>❑ 4= I use male condoms<br>❑ 5= I tried using it, but did not like the female condom<br>❑ 6= My partner did not like using the female condom<br>❑ 7= Because I am married<br>❑ 8= Because I am/we are faithful<br>❑ 9= I am frightened to try it<br>❑ 10= It interrupts sex/ruins the moment<br>❑ 11= Don't think it's necessary<br>❑ 12= I have never had sex/not sexually active<br>❑ 77= Other, *SPECIFY*___________________________	

**6.7 Now I'd like to ask you a series of questions to get a sense of how you feel about the female condom. Even if you have never used the female condom, give me the answer that best reflects how you feel. For each statement I read, please tell me whether you strongly agree, somewhat agree, somewhat disagree, or strongly disagree**.						
SHOW OPTION CARD 1		Strongly Agree	Somewhat Agree	Somewhat Disagree	Strongly Disagree	Don't Know
**a.**	**Female condoms make sex better for women.**	❑ 1	❑ 2	❑ 3	❑ 4	❑ 88
**b.**	**Female condoms feel more natural than regular male condoms.**	❑ 1	❑ 2	❑ 3	❑ 4	❑ 88
**c.**	**Female condoms make sex last long.**	❑ 1	❑ 2	❑ 3	❑ 4	❑ 88
**d.**	**Female condoms are better than male condoms.**	❑ 1	❑ 2	❑ 3	❑ 4	❑ 88
**e.**	**Female condoms are weird.**	❑ 1	❑ 2	❑ 3	❑ 4	❑ 88
**f.**	**Female condoms are inconvenient.**	❑ 1	❑ 2	❑ 3	❑ 4	❑ 88
**g.**	**Female condoms are messy.**	❑ 1	❑ 2	❑ 3	❑ 4	❑ 88
**h.**	**Having part of the female condom outside the vagina is unappealing/turn-off**	❑ 1	❑ 2	❑ 3	❑ 4	❑ 88
**i.**	**Female condoms offer better protection against unintended pregnancy than male condoms do.**	❑ 1	❑ 2	❑ 3	❑ 4	❑ 88
**j.**	**Female condoms offer better protection against sexually transmitted diseases than male condoms do.**	❑ 1	❑ 2	❑ 3	❑ 4	❑ 88
**k.**	**Female condoms are stronger than male condoms.**	❑ 1	❑ 2	❑ 3	❑ 4	❑ 88
**l.**	**The female condom takes too long to put in.**	❑ 1	❑ 2	❑ 3	❑ 4	❑ 88
**m.**	**It is hard to carry female condoms in a purse because of their size.**	❑ 1	❑ 2	❑ 3	❑ 4	❑ 88
**n.**	**Female condoms put the woman in charge.**	❑ 1	❑ 2	❑ 3	❑ 4	❑ 88
**o.**	**The female condom provides women another contraceptive choice.**	❑ 1	❑ 2	❑ 3	❑ 4	❑ 88
**p.**	**The female condom provides women another choice to protect themselves against HIV and other sexually transmitted diseases.**	❑ 1	❑ 2	❑ 3	❑ 4	❑ 88
**q.**	**Sex doesn't feel as good when you use a female condom.**	❑ 1	❑ 2	❑ 3	❑ 4	❑ 88
**r.**	**Female condoms make it hard for a woman to have an orgasm (cum).**	❑ 1	❑ 2	❑ 3	❑ 4	❑ 88
**s.**	**Female condoms make it hard for a man to have an orgasm (cum).**	❑ 1	❑ 2	❑ 3	❑ 4	❑ 88
**t.**	**Female condoms take all the fun out of sex.**	❑ 1	❑ 2	❑ 3	❑ 4	❑ 88
**u.**	**You don't like the idea or thought of putting the female condom inside yourself.**	❑ 1	❑ 2	❑ 3	❑ 4	❑ 88
**v.**	**You don't like the idea or thought of having to touch yourself to put the female condom in.**	❑ 1	❑ 2	❑ 3	❑ 4	❑ 88
**w.**	**You don't like the idea or thought of having to use your finger to put the female condom in.**	❑ 1	❑ 2	❑ 3	❑ 4	❑ 88
**x.**	**If a woman wants to use a female condom, her partner might think she was having sex with someone else.**	❑ 1	❑ 2	❑ 3	❑ 4	❑ 88

This is the end of our interview. Thank you so much for sharing your ideas with me. Do you have any questions, or is there anything that you would like to add before we end? If you have further thoughts about any of the issues we discussed today, please call Prof. Jenni Smit, the Principal Investigator of the study. Her contact details are on the information sheet/consent form that you were given.
