# Supplementary material for: Lessons from the evaluation of the South African National Female Condom Programme
Source: PLoS One. 2020 Aug 13;15(8):e0236984. doi: 10.1371/journal.pone.0236984 (PMC7425948; doi:10.1371/journal.pone.0236984)
Supplement: S3 File — (PDF) [file pone.0236984.s003.pdf]

PIN ❑❑❑ SITE NUMBER ❑❑❑❑❑

Evaluation of the National South African Female Condom Programme: Investigating Factors Associated with Uptake and Sustained Use
ANONYMOUS FEMALE CONDOM SURVEY

Good day, MatCH Research (Maternal, Adolescent and Child Health Research) of the University of the Witwatersrand and the HIV Centre at Columbia University from the USA would like to invite you to participate in an anonymous survey about female condoms. We are conducting a national evaluation of South Africa's female condom programme. We are interested in what clients in this clinic know about female condoms. This survey should only take a few minutes of your time. If you are willing to complete the survey, the clerk will give you a pen and a clipboard. Please follow the instructions for each question.

When you finish the survey, please put the survey in the box marked "Anonymous Female Condom Survey" on the reception desk. Do not put your name on the questionnaire. No one in the clinic apart from the MatCH Research staff will look at the surveys. If there are any questions that you do not want to answer, you can leave them blank.

1. How old are you?

WRITE YOUR AGE IN YEARS IN THE BOX ☐☐

PIN ❑❑❑ SITE NUMBER ❑❑❑❑❑

For each question below, please put an X in the box that best fits your answer.

2. Are you male or female?

❑ Male ❑ Female

3. Have you heard about a condom that women can use called the female condom?

❑ 1= Yes → **GO TO QUESTION 4**
❑ 2= No → *If you have <u>never heard</u> of female condoms, you do not need to complete the rest of the survey. Thank you and please put your survey in the box even if you stop at this question.*

4. Are female condoms available at this facility/site?

❑ 1= Yes → **GO TO QUESTION 5**
❑ 2= No → **GO TO QUESTION 7**
❑ 88= Don't know

5. Have you ever been offered female condoms at this facility/site?

❑ 1= Yes → **GO TO QUESTION 6**
❑ 2= No → **GO TO QUESTION 7**

6. If you were offered female condoms, did you take them?

❑ 1= Yes
❑ 2= No
→ **GO TO QUESTION 7**

7. Have you ever used a female condom with any partner?

❑ 1= Yes → **GO TO QUESTION 8**
❑ 2= No
❑ 3= Don't know
→ **GO TO QUESTION 10**

8. [Do you/did you] use female condoms for preventing sexually transmitted infections, including HIV, or [do you/did you] use them for preventing pregnancy, or for preventing both STIs/HIV and pregnancy?

- ❑ 1= For preventing STIs/HIV only
- ❑ 2= For preventing pregnancy only
- ❑ 3= For preventing STIs/HIV and pregnancy

→ **GO TO QUESTION 9**

9. If you have ever used a female condom, how often do you use female condoms with your <u>current</u> partner/s?

- ❑ 1= Never use female condoms → **GO TO QUESTION 10**
- ❑ 2= Sometimes use female condoms
- ❑ 3= Often use female condoms
- ❑ 4= Always use female condoms
- ❑ 5= No current partner

→ **GO TO END**

10. IF NEVER used a female condom, or NEVER used with current partner, please give your reason/s by ticking in the boxes below.
PLEASE PUT AN ***X*** *IN THE BOX. YOU CAN CHOOSE MORE THAN 1 ANSWER.*

- ❑ 1= Don't know where to get female condoms
- ❑ 2= They are not available here/out-of-stock
- ❑ 3= Partner will object
- ❑ 4= Use other contraceptive
- ❑ 5= I tried using it but did not like the female condom
- ❑ 6= My partner did not like using the female condom
- ❑ 7= Because I am married
- ❑ 8= Because I am faithful
- ❑ 9= I am frightened to try it
- ❑ 10= It interrupts sex/ruins the moment
- ❑ 11= Don't think it's necessary

❑ 12= My partner did not like using it.
❑ 13= Never been sexually active
❑ 77=Other (*PLEASE SPECIFY*)___________________________

THANK YOU FOR COMPLETING THIS QUESTIONNAIRE.
PLEASE PUT IT IN THE BOX AT RECEPTION.
