# Supplementary material for: Lessons from the evaluation of the South African National Female Condom Programme
Source: PLoS One. 2020 Aug 13;15(8):e0236984. doi: 10.1371/journal.pone.0236984 (PMC7425948; doi:10.1371/journal.pone.0236984)
Supplement: S4 File — (PDF) [file pone.0236984.s004.pdf]

EXIT INTERVIEW WITH FEMALE CLIENTS	
**INSTRUCTIONS TO INTERVIEWER:** For each of the questions listed below, mark the code that best represents the participant's response, or write the participant's own words (verbatim) for the open-ended questions.	
Facility name/section	
Facility code	[___|___]___|___]___]
Client ID	[__|__|__]
Client FC user type code	❑ 1= Ex-user<br>❑ 2= Sometimes user<br>❑ 3= Regular user
Interviewer code	|__|__]
Date of interview (Date/month/year)	___ ___/___ ___/ ___ ___
Interview start time	_____:_____<br>H H : M M
Interview stop time	_____:_____<br>H H : M M
Results codes	❑ 1= Complete<br>❑ 2= Partly complete<br>❑ 3= Refused to continue
**CHECKED BY:** ………………..…………………………………………………..<br>**DATE CHECKED:** …………………………………………………………....<br>**DATA ENTRY BY:** ……………………………………………………………..<br>**DATA ENTRY DATE:** …………………………………………………………	

**1.0 BACKGROUND INFORMATION**<br><br>**First, I would like to ask you some questions about your visit to the clinic today and your background. Some of the questions that I am going to ask you may be sensitive, but remember, our conversation will be confidential.**		
**1.1**	**What was/were the reason/s for your visit today to this facility/site?**<br>*MARK ALL THAT CLIENT MENTIONS*	❑ 1= Wanted FP<br>❑ 2= Had STI symptoms of discharge, ulcer, or genital pain<br>❑ 3= Came for HIV care (wellness)<br>❑ 4= Came for ART follow-up<br>❑ 5= To have HIV testing and counselling<br>❑ 6= To access SGBV counselling and related services<br>❑ 7= For CTOP services and/or referral<br>❑ 8= General women's health service, e.g. cervical cancer screening/follow-up<br>❑ 9= Wanted condoms<br>❑ -77= Other,<br>*SPECIFY*____<br>____
**1.2**	**Why did you choose to come to this facility for the services you just mentioned?**<br>*MARK ALL THAT CLIENT MENTIONS*	❑ 1= Referred here by another facility<br>❑ 2= Closest facility<br>❑ 3= Only facility I know<br>❑ 4= I like the service I get here<br>❑ 5= I like the providers here<br>❑ 6= Provides a wide range of services<br>❑ -77= Other,<br>*SPECIFY*____<br>____
**1.3**	**How old are you?**	Age in completed years _________
**1.4**	**What is your highest level of education completed?**	❑ 1= None<br>❑ 2= Primary incomplete<br>❑ 3= Primary complete<br>❑ 4= Secondary incomplete<br>❑ 5= Secondary complete<br>❑ 6= Any tertiary level
**1.5**	**What is your religion?**	❑ 1= Christian, Anglican, Catholic, Methodist, Lutheran etc.<br>❑ 2= Zionist<br>❑ 3= Muslim<br>❑ 4= Hindu<br>❑ 5= Apostolic church<br>❑ 6= Atheist/agnostic<br>❑ 7= Ancestral worship<br>❑ -77= Other,<br>*SPECIFY*____

1.6	**Who do you live with most of the time?**<br>*IF YOU ARE A STUDENT, WHERE DO YOU LIVE WHEN NOT IN RESIDENCE?*	❑ 1 = With my partner<br>❑ 2 = Family/relatives<br>❑ 3 = Friends<br>❑ 4 = I live on my own<br>❑ 5 = Somewhere else,<br>*SPECIFY*____<br>____
1.7	**What is your current employment situation? By employment, I mean any type of work that provides you with money, whether you are self-employed, employed by someone else, whether you work in the formal or informal sector.**<br><br>*MARK ALL THAT APPLY*	❑ 1= Housewife, homemaker, not looking for work<br>❑ 2= Housewife, homemaker, looking for work<br>❑ 3= Unemployed, looking for work<br>❑ 4= Unemployed, not looking for work<br>❑ 5= Informal sector, not looking for permanent work<br>❑ 6= Student/pupil/learner<br>❑ 7= Self-employed - full time (40 hours or more per week)<br>❑ 8= Self-employed - part time (< than 40 hours per week)<br>❑ 9= Employed p/t(If none of the above.(< 40 hrs/ week)<br>❑ 10= Employed full time (40 hours or more per week)<br>❑ -77= Other,<br>*SPECIFY*____
1.8	**Where does money come from to pay towards your household expenses (food, bills, etc, school fees, etc.)?**<br><br>*READ ALL RESPONSES IN LIST AND MARK ALL THAT APPLY*	❑ 1= Self<br>❑ 2= My partner/s<br>❑ 3= Family members/relatives<br>❑ 4= Friend/s<br>❑ 5= Government grants<br>❑ -77= Other, *SPECIFY*<br>______________________________________

**2.0 FAMILY PLANNING**<br>**Now I am going to ask you some questions about family planning. Some of the questions may be sensitive, but remember, our conversation will be confidential.**		
2.1	**Have you ever been pregnant?**	❑ 0= No *IF NO SKIP TO Q2.4*<br>❑ 1= Yes
2.2	**Was your last pregnancy planned?**	❑ 0= No<br>❑ 1= Yes
2.3	**How many living children do you have?**	______(No. of living children)
2.4	**Do you want to become pregnant in the next year?**	❑ 0= No<br>❑ 1= Yes<br>❑ 88= Not sure

2.5 **I am now going to ask you about different methods that you may have heard about or used that can prevent pregnancy, STIs/HIV, or both.** *PROMPT*
NOTE ALL PARTICIPANTS SHOULD HAVE EVER USED FC

	Method	Ever heard of	Ever used	Current use (used in last 3 months)	At last sex
a.	**2-month injectable (NET-EN)**	❑ 0= No<br>❑ 1= Yes	❑ 0= No<br>❑ 1= Yes	❑ 0= No<br>❑ 1= Yes	
b.	**3-month injectable (Depo)**	❑ 0= No<br>❑ 1= Yes	❑ 0= No<br>❑ 1= Yes	❑ 0= No<br>❑ 1= Yes	
c.	**Contraceptive pills-combined**	❑ 0= No<br>❑ 1= Yes	❑ 0= No<br>❑ 1= Yes	❑ 0= No<br>❑ 1= Yes	
d.	**Progestin-only contraceptive pills (POPs)**	❑ 0= No<br>❑ 1= Yes	❑ 0= No<br>❑ 1= Yes	❑ 0= No<br>❑ 1= Yes	
e.	**IUD**	❑ 0= No<br>❑ 1= Yes	❑ 0= No<br>❑ 1= Yes	❑ 0= No<br>❑ 1= Yes	
f.	**Hormonal implant**	❑ 0= No<br>❑ 1= Yes	❑ 0= No<br>❑ 1= Yes	❑ 0= No<br>❑ 1= Yes	
g.	**Sterilization**	❑ 0= No<br>❑ 1= Yes	❑ 0= No<br>❑ 1= Yes	❑ 0= No<br>❑ 1= Yes	
h.	**Female condoms**	❑ 0= No<br>❑ 1= Yes	❑ 0= No<br>❑ 1= Yes	❑ 0= No<br>❑ 1= Yes	❑ 0= No<br>❑ 1= Yes
i.	**Male condoms**	❑ 0= No<br>❑ 1= Yes	❑ 0= No<br>❑ 1= Yes	❑ 0= No<br>❑ 1= Yes	❑ 0= No<br>❑ 1= Yes
j.	**Withdrawal**	❑ 0= No<br>❑ 1= Yes	❑ 0= No<br>❑ 1= Yes	❑ 0= No<br>❑ 1= Yes	❑ 0= No<br>❑ 1= Yes
k.	**Emergency contraception**	❑ 0= No<br>❑ 1= Yes	❑ 0= No<br>❑ 1= Yes	❑ 0= No<br>❑ 1= Yes	❑ 0= No<br>❑ 1= Yes
l.	**LAM (breastfeeding)**	❑ 0= No<br>❑ 1= Yes	❑ 0= No<br>❑ 1= Yes	❑ 0= No<br>❑ 1= Yes	❑ 0= No<br>❑ 1= Yes
m.	**Fertility awareness-based/ calendar-based method/NFP**	❑ 0= No<br>❑ 1= Yes	❑ 0= No<br>❑ 1= Yes	❑ 0= No<br>❑ 1= Yes	❑ 0= No<br>❑ 1= Yes
n.	**Other,** ***SPECIFY***_____________	_________	_______	__________	_______

**How confident are you that…**<br>*SHOW OPTION CARD 1*		**Very sure**	**Somewhat sure**	**Somewhat unsure**	**Very unsure**
**2.6**	**You can talk to your partner about contraception?**	❑ 1	❑ 2	❑ 3	❑ 4
**2.7**	**You could use contraception even if your partner does not want you to?**	❑ 1	❑ 2	❑ 3	❑ 4

3.0 FEMALE CONDOM USE
Now I am going to ask you some questions about female condoms.
Some of the questions may be sensitive, but remember, our conversation will be confidential.

3.1 **Can you remember how you first heard about female condoms?**
MARK 'YES', 'NO', OR 'CAN'T REMEMBER'. GIVE ONE ANSWER ONLY. DO NOT PROMPT

	First heard	At this facility/site	At another facility/site	Outside facility/site
a.	A provider /counselor first told me about FCs today	❑ 0= No<br>❑ 1= Yes<br>❑ 2= Can't remember	❑ 0= No<br>❑ 1= Yes<br>❑ 2= Can't remember	❑ 0= No<br>❑ 1= Yes<br>❑ 2= Can't remember
b.	A provider told me about FCs before today	❑ 0= No<br>❑ 1= Yes<br>❑ 2= Can't remember	❑ 0= No<br>❑ 1= Yes<br>❑ 2= Can't remember	❑ 0= No<br>❑ 1= Yes<br>❑ 2= Can't remember
c.	I saw a poster	❑ 0= No<br>❑ 1= Yes<br>❑ 2= Can't remember	❑ 0= No<br>❑ 1= Yes<br>❑ 2= Can't remember	❑ 0= No<br>❑ 1= Yes<br>❑ 2= Can't remember
d.	I read/got a leaflet/pamphlet	❑ 0= No<br>❑ 1= Yes<br>❑ 2= Can't remember	❑ 0= No<br>❑ 1= Yes<br>❑ 2= Can't remember	❑ 0= No<br>❑ 1= Yes<br>❑ 2= Can't remember
e.	Newspaper /magazine	❑ 0= No<br>❑ 1= Yes<br>❑ 2= Can't remember	❑ 0= No<br>❑ 1= Yes<br>❑ 2= Can't remember	❑ 0= No<br>❑ 1= Yes<br>❑ 2= Can't remember
f.	I heard on TV/radio	❑ 0= No<br>❑ 1= Yes<br>❑ 2= Can't remember	❑ 0= No<br>❑ 1= Yes<br>❑ 2= Can't remember	❑ 0= No<br>❑ 1= Yes<br>❑ 2= Can't remember
g.	A friend told me	❑ 0= No<br>❑ 1= Yes<br>❑ 2= Can't remember	❑ 0= No<br>❑ 1= Yes<br>❑ 2= Can't remember	❑ 0= No<br>❑ 1= Yes<br>❑ 2= Can't remember
h.	My partner told me	❑ 0= No<br>❑ 1= Yes<br>❑ 2= Can't remember	❑ 0= No<br>❑ 1= Yes<br>❑ 2= Can't remember	❑ 0= No<br>❑ 1= Yes<br>❑ 2= Can't remember
i.	Other, *SPECIFY*________	❑ 0= No<br>❑ 1= Yes<br>❑ 2= Can't remember	❑ 0= No<br>❑ 1= Yes<br>❑ 2= Can't remember	❑ 0= No<br>❑ 1= Yes<br>❑ 2=Can't remember

<table>
<tr><td>3.2</td><td>When was the <u>first</u> time you used a female condom?
IF LESS THAN 1 YEAR, WRITE <1</td><td>Years ago_____________</td><td></td></tr>
<tr><td>3.3</td><td colspan="2">Why did you <u>first</u> start using female condoms
DO NOT PROMPT

MARK ALL THE RESPONSES THAT APPLY.
<table>
<tr><th></th><th>Reason for taking the female condom</th><th>First use</th></tr>
<tr><td>a.</td><td>Just wanted to try one/no particular reason</td><td>❑</td></tr>
<tr><td>b.</td><td>I do not like male condoms</td><td>❑</td></tr>
<tr><td>c.</td><td>My partner would not use male condoms</td><td>❑</td></tr>
<tr><td>d.</td><td>My partner suggested we try them</td><td>❑</td></tr>
<tr><td>e.</td><td>A friend suggested I try them</td><td>❑</td></tr>
<tr><td>f.</td><td>A provider/counselor suggested I try them</td><td>❑</td></tr>
<tr><td>g.</td><td>I wanted to protect myself from STIs/HIV</td><td>❑</td></tr>
<tr><td>h.</td><td>I wanted to protect myself from pregnancy</td><td>❑</td></tr>
<tr><td>i.</td><td>Novelty of use/experiment</td><td>❑</td></tr>
<tr><td>j.</td><td>Other, SPECIFY_________</td><td>________
____
________</td></tr>
</table></td><td></td></tr>
<tr><td>3.4</td><td colspan="2">Who did you get your <u>first</u> FC from?
IF ANOTHER FACILITY/SITE, WRITE THE NAME, AND IF PUBLIC SECTOR/NGO ETC.

❑ 1= Provider/counselor this facility/site
❑ 2= Provider counselor another facility/site (NAME/TYPE)________________________
❑ 3= My partner
❑ 4= A friend
❑ 5= No one, I got one myself from a dispenser/box/[NAME PLACE]________________</td><td></td></tr>
<tr><td>3.5</td><td>Do you remember how many female condoms you were given/ you took the first time? ESTIMATE IF POSSIBLE</td><td>Number ________
❑ 88= Don’t know/can’t remember</td><td></td></tr>
<tr><td>3.6</td><td>In addition to the place you mentioned as the first place --------------. Did you/do you ever get a supply of FCs from other places?
MARK ALL THAT ARE MENTIONED</td><td>❑ 1= No, only this site
❑ 2= NGO site/s
❑ 3= Another public sector facility
❑ 4= Other SPECIFY__________________</td><td></td></tr>
<tr><td>3.7</td><td>IF PARTICIPANT’S RESPONSE TO Q3.4 WAS 3, SKIP TO Q3.13; IF 4 SKIP TO Q3.12; IF 5 SKIP TO Q3.11

IF RESPONSE TO Q3.4 WAS 1 OR 2 ASK-Thinking of the provider who gave you female condoms (Q3.4) did you ask for a female condom or did she/he offer you one to try? .</td><td>❑ 1= I was offered/given FCs
❑ 2= I asked for FCs
❑ 88= I can’t remember/not sure
❑ 77= Other,
SPECIFY____________________________
_____________________________________</td><td></td></tr>
</table>

3.8	**What did they explain to you/give you about how to use a female condom?**<br>*READ LIST AND MARK ALL THAT PARTICIPANT MENTIONS*<br><br>❑ 1= Explained how to use<br>❑ 2= Showed me an actual female condom<br>❑ 3= Gave me a demonstration with hand<br>❑ 4= Gave me a demonstration on a model<br>❑ 5= Gave me a leaflet/pamphlet to take home<br>❑ 6= Told me to come back if I had any problems<br>❑ 7= Answered my questions<br>❑ 8= They gave me no information<br>❑ 77= Other, *SPECIFY*______________________________<br>______________________________		
3.9	**Did you get any advice on how to introduce the female condom to your partner the first time you got them?**<br>*EVEN IF P DOES NOT HAVE A PARTNER NOW, P SHOULD ANSWER THIS QUESTION IN RELATION TO PARTNER WITH WHOM FC FIRST USED*	❑ 0= No<br>❑ 1= Yes	IF NO, GO TO Q3.12
3.10	**What advice did you get?** *MARK ALL THAT PARTICIPANT MENTIONS*	❑ 1= Tell him it means he does not have to use a MC<br>❑ 2= Tell him he will enjoy sex more<br>❑ 3= Show him the FC and the instructions<br>❑ 4= Explain to him how FC works<br>❑ 77= Other, *SPECIFY*____________________<br>____________________	ALL GO TO Q3.12
3.11	**You told me that you got your first FC from a dispenser/box. Where did you get information about how to use the FC?**	❑ 1= I took a leaflet/pamphlet<br>❑ 2= I used the instructions printed on the packet<br>❑ 3= Worked out how to use it myself	All GO TO Q3.12
3.12	**The first time you used the FC how did your partner react when you suggested use of the FC?**	❑ 1= He agreed to try it<br>❑ 2= He agreed after some persuasion/discussion<br>❑ 3= He refused to use it<br>❑ 4= He suggested it<br>❑ 5= Other *SPECIFY*____________	

**3.13**	**Did you feel you had enough information that you were confident in using the female condom the <u>first time</u> you used it?**	❑ 1= Yes<br>❑ 2= No	ALL GO TO Q3.14
**3.14**	**How many FCs did you use before you felt confident that you were using them correctly?**	Number________________<br>❑ 99= Never felt/don't feel confident	
**3.15**	**How many times have you <u>ever</u> used FCs?**	❑ 1= At least 10 times<br>❑ 2= At least 5 times, but less than 10<br>❑ 3= Between 2 and 4 times<br>❑ 4= Only ever used once	
**3.16**	**How effective do you think the female condom is at preventing HIV and other STIs? Would you say extremely effective, somewhat effective, somewhat ineffective, or extremely ineffective?**<br>*SHOW OPTION CARD 2*	❑ 1= Extremely ineffective<br>❑ 2= Somewhat ineffective<br>❑ 3= Somewhat effective<br>❑ 4= Extremely effective<br>❑ 88= Don't know	
**3.17**	**How effective do you think the female condom is at preventing pregnancy? Would you say extremely effective, somewhat effective, somewhat ineffective, or extremely ineffective?**<br>*SHOW OPTION CARD 2*	❑ 1= Extremely ineffective<br>❑ 2= Somewhat ineffective<br>❑ 3= Somewhat effective<br>❑ 4= Extremely effective<br>❑ 88= Don't know	
**3.18**	**If you had the option of using a male or a female condom, which would you choose?**	❑ 1= Female condom<br>❑ 2= Male condom<br>❑ 3= Alternate between male and female condoms, like them both equally<br>❑ 4= Neither, don't like either type	
**3.19**	**Can you tell me your main reason for this choice?**<br>*ASK FOR ONE MAIN REASON*	❑ 1= I prefer the female condom<br>❑ 2= I prefer the male condom<br>❑ 3= My partner prefers the male condom<br>❑ 4= My partner prefers the female condom.<br>❑ 5= Other_____________	

4.0 THESE QUESTIONS ARE FOR FEMALE CONDOM EX-USERS - I.E NOT USED IN THE LAST 3 MONTHS. *IF REGULAR OR SOMETIMES FC USERS GO TO SECTION 5*
Now I am going to ask you some questions about why you stopped using the female condom. This is okay, we are just interested in understanding why you did not use the female condom.
Some of the questions may be sensitive, but remember, our conversation will be confidential.

**4.1**	**When did you last use a female condom?**<br>*IF LESS THAN A YEAR, WRITE MONTHS. IF MORE, WRITE YEARS*	________________months ago<br>___________________years ago

4.2 Why did you stop using female condoms? If your partner was the reason you stopped using the female condom, tell us about that?
INDICATE ALL REASONS GIVEN FOR BOTH PARTICIPANT AND HER PARTNER. MARK ALL THAT APPLY IN THE 'WOMAN' AND 'PARTNER' COLUMNS- *DO NOT PROMPT*

	Reason	A. Woman	B. Partner
a.	**No longer with current partner- no sexual partner**	❑ 1= Yes	
b.	**Partner refused to use it**		❑ 1= Yes
c.	**The FC broke when I opened the packet**	❑ 1= Yes	
d.	**Difficulty in handling it before insertion**	❑ 1= Yes	
e.	**FC broke/ripped when I tried to insert it.**	❑ 1= Yes	
f.	**Unsure how deeply to insert it**	❑ 1= Yes	
g.	**Difficult to insert**	❑ 1= Yes	
h.	**Not sure if FC was in the right place**	❑ 1= Yes	
i.	**Outer ring was bothersome**	❑ 1= Yes	
j.	**Inner ring was uncomfortable**	❑ 1= Yes	❑ 1= Yes
k.	**Worried it might get stuck inside vagina**	❑ 1= Yes	❑ 1= Yes
l.	**Unattractive/a turn-off/put off sex**	❑ 1= Yes	❑ 1= Yes
m.	**Prefer male condom**	❑ 1= Yes	❑ 1= Yes
n.	**Other, *SPECIFY***	❑ 1= Yes, SPECIFY<br>_______________	❑ 1= Yes, SPECIFY<br>_______________

**4.3**	***IF PARTNER REFUSED TO USE FC (b above), ASK***<br>**If your partner had liked/was willing to use the FC, would you have continued to use it?**	❑ 0= No<br>❑ 1= Yes
**4.4**	**Do you think you will use the female condom in the future?**	❑ 0= No<br>❑ 1= Yes<br>❑ 2= depends on partner<br>❑ 88= Don't know

**5.0 PEER NORMS** *SHOW OPTION CARD 3 FOR Q5.5 & Q5.6*		
**5.1**	**As far as you know, have any of your friends used a female condom?**	❑ 0= No<br>❑ 1= Yes<br>❑ 88= Don't know
**5.2**	**Do any of your friends think that it is important to use the female condom for protection against HIV and other STIs?**	❑ 0= No<br>❑ 1= Yes<br>❑ 88= Don't know
**5.3**	**Do any of your friends think that it is important to use the female condom for protection against pregnancy?**	❑ 0= No<br>❑ 1= Yes<br>❑ 88= Don't know
**5.4**	**Have you discussed the female condom with any of your friends?**	❑ 0= No<br>❑ 1= Yes
**5.5**	**In terms of protecting yourself against pregnancy or HIV and other sexually transmitted infections, how important is it to do what your <u>women friends</u> do?**<br><br>**Would you say 'very important', 'important', 'unimportant', 'very unimportant'?** *OPTION CARD 3*	❑ 1= Very unimportant<br>❑ 2= Unimportant<br>❑ 3= Important<br>❑ 4= Very important<br>❑ 88= Don't know/not sure
**5.6**	**In terms of protecting yourself against pregnancy or HIV and other sexually transmitted infections, how important is it to do what <u>your partner</u> thinks you should do?**<br><br>**Would you say 'very important', 'important', 'unimportant', or 'very unimportant'?** *OPTION CARD 3*	❑ 1= Very unimportant<br>❑ 2= Unimportant<br>❑ 3= Important<br>❑ 4= Very important<br>❑ 88= Don't know/not sure

**6.0 SEXUAL RELATIONSHIPS AND USE OF THE FEMALE CONDOM**<br>*THIS SECTION IS FOR ALL WOMEN*			
**6.1**	**I would now like to ask you about your present relationship or relationships.**<br><br>**Are you currently**:<br>*INTERVIEWER TO READ RESPONSES AND MARK ALL THAT PARTICIPANT MENTIONS*	❑ 1= Married, living together<br>❑ 2= Married, living apart<br>❑ 3= Not married, living with partner<br>❑ 4= Regular visiting partner/s<br>❑ 5= Casual partner/s<br>❑ 6= Separated from marital partner<br>❑ 7= Divorced<br>❑ 77= Other, *SPECIFY*___________	
**6.2**	**Describe your current sexual relationship status. Do you currently have one main/regular sexual partner, or do you have casual partners, or both main and casual partners?** *PROMPT AND MARK ONE*	❑ 1= One regular/primary partner<br>❑ 2= Casual partners only<br>❑ 3= Both regular partner and casual partners	If 2 OR 3, GO TO Q6.3<br>IF 1, SKIP Q6.4
**6.3**	**How many partners do you have altogether at this time?**	Casual number of partners______<br>Regular number of partners______	

We would like to ask you some questions about your most recent partners. Think about the sexual partners you have had in the past 2 months. *USE A CALENDAR TO ESTABLISH THE 2-MONTH PERIOD*. **Can you remember what was going on in your life in the past 2 months?** *WAIT FOR RESPONSES AND PROBE IF NECESSARY*.
COMPLETE THE COLUMN MARKED ***A. PARTNER 1 MOST RECENT*** *FOR THE MOST RECENT PARTNER WITH WHOM PARTICIPANT HAD SEX WITH IN THE LAST 2 MONTHS. THIS IS REGARDLESS OF WHETHER OR NOT PARTICIPANT IS STILL HAVING SEX WITH THAT PARTNER.*

You told me in Q6.2/6.3 that you had [*NUMBER*] partners. I would like to ask about your/ the three most recent partners you last had sex with. If you don't mind, can I write down their initials so I am sure I am asking you about the correct partner?

		A. Partner 1<br>Most recent	B. Partner 2<br>Next most recent	C. Partner 3<br>Next most recent
**6.4 First, will you tell me a nickname or the initials of your/each of the partners you have had sex with in the past 2 months, starting with your most recent partner.**<br>*PROBE FOR NEXT MOST RECENT PARTNER, ETC.*		________<br>**(Initials)**	________<br>**(Initials)**	________<br>**(Initials)**
**6.5**	**How would you describe this partner?**	❑ 1= Regular Boyfriend<br>❑ 2= Live in partner<br>❑ 3= Husband<br>❑ 4= Other partner	❑ 1= Regular boyfriend<br>❑ 2= Live in partner<br>❑ 3= Husband<br>❑ 4= Other partner	❑ 1= Regular boyfriend<br>❑ 2= Live in Partner<br>❑ 3= Husband<br>❑ 4= Other partner
**6.6**	**Do you consider** [*NAME*] **to be your main partner?**	❑ 0= Not MP<br>❑ 1= MP	❑ 0= Not MP<br>❑ 1= MP	❑ 0= Not MP<br>❑ 1= MP
**6.7**	**How old is** [*NAME*]**?**	Years___<br>❑ 88= Don't know	Years___<br>❑ 88= Don't know	Years___<br>❑ 88= Don't know
**6.8**	**Are you partially or fully financially dependent on** [*NAME*]**?**	❑ 0= No<br>❑ 1= Yes, partially<br>❑ 2= Yes, fully	❑ 0= No<br>❑ 1= Yes, partially<br>❑ 2= Yes, fully	❑ 0= No<br>❑ 1= Yes, partially<br>❑ 2= Yes, fully
**6.9**	**During the past 2 months, about how many times did you have vaginal sex with** [*NAME*] *EACH* [*DAY, WEEK, MONTH*]*?*<br><br>*PROBE FOR TIMES IN THE PAST 2 MONTHS WHEN THIS PATTERN DID NOT APPLY (PARTNER AWAY, ILL, OR P HAD PERIOD).*<br><br>*SUBTRACT FROM THE TOTAL THE NUMBER OF*	*COMPLETE ONE*<br>_____ /DAY x 30 = ___ ___ occasions<br><br>____/WEEK x 8 = ___ ___ occasions<br><br>_____/MONTH x 2 =<br>___ __ occasions<br><br>_____/LAST 2 MONTHS	*COMPLETE ONE*<br>_____ /DAY x 30 = ___ ___ ___ occasions<br><br>____/WEEK x 8 = ___ ___ occasions<br><br>_____/MONTH x 2=<br>___ ___ occasions<br><br>_____/LAST 2 MONTHS<br>x 1=___ ___occasions	*COMPLETE ONE*<br>_____ /DAY x 30 = ___ ___ ___ occasions<br><br>___/WEEK x 8 = ___ ___ occasions<br><br>____/MONTH x 2=<br>___ ___ occasions<br><br>_____/LAST 2 MONTHS<br>x 1= ___ ___ occasions

	OCCASIONS THAT WOULD HAVE OCCURRED DURING THAT TIME	x 1=____ ___ occasions		
**6.10**	**How many of those** [# *VAG SEX*] **times did your partner use a male condom?**	___ ___ ___<br># times male condom past 2 months	___ ___ ___<br># times male condom past 2 months	___ ___ ___<br># times male condom past 2 months
**6.11**	*SKIP TO 6.12 IF FC EX-USER*<br>**How many of those** [# *VAG SEX*] **times did your partner use a female condom?**	___ ___ ___<br># times female condom past month	___ ___ ___<br># times female condom past month	___ ___ ___<br># times female condom past month
**6.12**	**IF you have ever used a FC with any of your current partners, how did they react when you first suggested use?**<br>*IF NOT USED WITH ANY CURRENT PARTNER SKIP TO 6.14*	❑ 1= He agreed to try it<br>❑ 2= He agreed after some persuasion/discussion<br>❑ 3= He refused to use it<br>❑ 4= He suggested it<br>❑ 5= Other, *SPECIFY*____________	❑ 1= He agreed to try it<br>❑ 2= He agreed after some persuasion /discussion<br>❑ 3= He refused to use it<br>❑ 4= He suggested it<br>❑ 5= Other, *SPECIFY*________	❑ 1= He agreed to try it<br>❑ 2= He agreed after some persuasion/discussion<br>❑ 3= He refused to use it<br>❑ 4= He suggested it<br>❑ 5= Other, *SPECIFY*__________
**6.13**	**The last time you had vaginal intercourse, with** [*NAME*]**. Did you use a male condom, a female condom or no condom at all?**<br>*VERIFY Q2.5*	❑ 0= No condom<br>❑ 1= Male condom<br>❑ 2= Female condom	❑ 0= No condom<br>❑ 1= Male condom<br>❑ 2= Female condom	❑ 0= No condom<br>❑ 1= Male condom<br>❑ 2= Female condom
**6.14**	**Considering what you know or have heard about the male condom, how *(do you/would you)* feel about using it?**<br>*SHOW OPTION CARD* 4	❑ 1= Extremely negative<br>❑ 2= Somewhat negative<br>❑ 3= Neutral<br>❑ 4= Somewhat positive<br>❑ 5= Extremely positive<br>❑ 88= Don't know	❑ 1= Extremely negative<br>❑ 2= Somewhat negative<br>❑ 3= Neutral<br>❑ 4= Somewhat positive<br>❑ 5= Extremely positive<br>❑ 88= Don't know	❑ 1= Extremely negative<br>❑ 2= Somewhat negative<br>❑ 3= Neutral<br>❑ 4= Somewhat positive<br>❑ 5= Extremely positive<br>❑ 88= Don't know
**6.15**	**How about your partner? How does he feel about using the male condom?**<br>*OPTION CARD 4*<br>IF P SAYS "HE LEAVES IT TO ME", CODE RESPONSE AS 'NEUTRAL'	❑ 1= Extremely negative<br>❑ 2= Somewhat negative<br>❑ 3= Neutral<br>❑ 4= Somewhat positive<br>❑ 5= Extremely positive<br>❑ 88= Don't know	❑ 1= Extremely negative<br>❑ 2= Somewhat negative<br>❑ 3= Neutral<br>❑ 4= Somewhat positive<br>❑ 5= Extremely positive<br>❑ 88= Don't know	❑ 1= Extremely negative<br>❑ 2= Somewhat negative<br>❑ 3= Neutral<br>❑ 4= Somewhat positive<br>❑ 5= Extremely positive<br>❑ 88= Don't know

**6.16**	**Considering what you know or have heard about the female condom, how *(do you/would you)* feel about using it?**<br><br>*OPTION CARD* 4	❑ 1= Extremely negative<br>❑ 2= Somewhat negative<br>❑ 3= Neutral<br>❑ 4= Somewhat positive<br>❑ 5= Extremely positive<br>❑ 88= Don't know	❑ 1= Extremely negative<br>❑ 2= Somewhat negative<br>❑ 3= Neutral<br>❑ 4= Somewhat positive<br>❑ 5= Extremely positive<br>❑ 88= Don't know	❑ 1= Extremely negative<br>❑ 2= Somewhat negative<br>❑ 3= Neutral<br>❑ 4= Somewhat positive<br>❑ 5= Extremely positive<br>❑ 88= Don't know
**6.17**	**How do you think** [*NAME*] **feels about using the female condom with you?**<br><br>*OPTION CARD 4*	❑ 1= Extremely negative<br>❑ 2= Somewhat negative<br>❑ 3= Neutral<br>❑ 4= Somewhat positive<br>❑ 5= Extremely positive<br>❑ 88= Don't know	❑ 1= Extremely negative<br>❑ 2= Somewhat negative<br>❑ 3= Neutral<br>❑ 4= Somewhat positive<br>❑ 5= Extremely positive<br>❑ 88= Don't know	❑ 1= Extremely negative<br>❑ 2= Somewhat negative<br>❑ 3= Neutral<br>❑ 4= Somewhat positive<br>❑ 5= Extremely positive<br>❑ 88= Don't know
**6.18**	**When was the last time you were afraid to ask** [*NAME*] **to use condoms because he might get angry?**	❑ 1= Never<br>❑ 2= In the past 30 days<br>❑ 3= I have done this, but not in the past 30 days	❑ 1= Never<br>❑ 2= In the past 30 days<br>❑ 3= I have done this, but not in the past 30 days	❑ 1= Never<br>❑ 2= In the past 30 days<br>❑ 3= I have done this, but not in the past 30 days
**6.19**	**How likely is it that you will use a condom – either a male or female condom – EVERY SINGLE TIME you have vaginal sex in the next month with** [*NAME*]**?**<br><br>*OPTION CARD 5*	❑ 1= Very unlikely<br>❑ 2= Unlikely<br>❑ 3= Likely<br>❑ 4= Very likely<br>❑ 88= Don't know	❑ 1= Very unlikely<br>❑ 2= Unlikely<br>❑ 3= Likely<br>❑ 4= Very likely<br>❑ 88= Don't know	❑ 1= Very unlikely<br>❑ 2= Unlikely<br>❑ 3= Likely<br>❑ 4= Very likely<br>❑ 88= Don't know

**7.0 CURRENT FC USERS ONLY IF EX-USER GO TO SECTION 8.0**			
**7.1**	**In general, how do you decide whether to use a male or a female condom when you have sex?**<br>*CIRCLE ALL THAT APPLY*	❑ 1= Depends on what condom is available<br>❑ 2= I like to have a choice/variety<br>❑ 3= My partner/s usually decide<br>❑ 4= I usually decide<br>❑ 5= Other, *SPECIFY*________________	
**7.2**	**Since you started using FCs, are you using condoms when you have sex (either male or female) more, less, or about the same?**	❑ 1= Using condoms more since started using FCs<br>❑ 2= Using condoms about the same<br>❑ 3= Using condoms less	

**7.3**	**Did you get any female or male condoms during your visit today?**	❑ 1= Yes<br>❑ 2= No	If 1 GO TO Q7.4;<br>IF 2 GO TO Q 7.5
**7.4**	**Where did you get the condoms from?**<br>*MARK ALL THAT APPLY*	❑ 1= FCs from provider<br>❑ 2= FCs from dispenser or box<br>❑ 3= MCs from provider<br>❑ 4= MCs from dispenser	
**7.5**	**If you did not get any female condoms today, where did you get your last (most recent) supply of female condoms?**	❑ 1= This facility<br>❑ 2= Another facility/site (*NAME*)_________<br>❑ 3= My partner gets them from, *SPECIFY*__________<br>❑ 4= Friend<br>❑ 77= Other, *SPECIFY* __________	
**7.6**	**How many FCs did you get today/ at your last (most recent) supply?**<br>*IF SHE GOT HER MOST RECENT SUPPLY TODAY, USE THIS NUMBER*	Number_________	
**7.7**	**Does your partner/s ever get/how often does he get female condoms?**	❑ 1= No, my partner never gets them<br>❑ 2= Yes, my partner sometimes gets them<br>❑ 3= Yes, my partner always gets them	

8.0 FEMALE CONDOM KNOWLEDGE AND ATTITUDES

Now I'd like to ask you a series of questions to get a sense of how you might feel about the female condom. For each statement I read, please tell me whether you strongly agree, somewhat agree, somewhat disagree or strongly disagree. *DO NOT MENTION 'DON'T KNOW' OPTION, BUT IF PARTICIPANT SAYS 'DON'T KNOW', MARK THAT OPTION*. *SHOW OPTION CARD 6*

		Strongly Agree	Somewhat Agree	Somewhat Disagree	Strongly Disagree	Don't Know
**8.1**	**Female condoms make sex better for women.**	❑ 1	❑ 2	❑ 3	❑ 4	❑ 88
**8.2**	**Female condoms feel more natural than regular male condoms.**	❑ 1	❑ 2	❑ 3	❑ 4	❑ 88
**8.3**	**Female condoms make sex last long.**	❑ 1	❑ 2	❑ 3	❑ 4	❑ 88
**8.4**	**Female condoms are better than male condoms.**	❑ 1	❑ 2	❑ 3	❑ 4	❑ 88
**8.5**	**Female condoms are weird.**	❑ 1	❑ 2	❑ 3	❑ 4	❑ 88
**8.6**	**Female condoms are inconvenient.**	❑ 1	❑ 2	❑ 3	❑ 4	❑ 88
**8.7**	**Female condoms are messy.**	❑ 1	❑ 2	❑ 3	❑ 4	❑ 88
**8.8**	**Having the outer part of the female condom visible on the vagina is unattractive/turn off.**	❑ 1	❑ 2	❑ 3	❑ 4	❑ 88

**8.9**	**Female condoms offer better protection against unintended pregnancy than male condoms do.**	❑ 1	❑ 2	❑ 3	❑ 4	❑ 88
**8.10**	**Female condoms offer better protection against sexually transmitted diseases than male condoms do.**	❑ 1	❑ 2	❑ 3	❑ 4	❑ 88
**8.11**	**Female condoms are stronger than male condoms.**	❑ 1	❑ 2	❑ 3	❑ 4	❑ 88
**8.12**	**The female condom takes too long to put in.**	❑ 1	❑ 2	❑ 3	❑ 4	❑ 88
**8.13**	**It is hard to carry female condoms in a purse because of their size.**	❑ 1	❑ 2	❑ 3	❑ 4	❑ 88
**8.14**	**Female condoms put the woman in charge.**	❑ 1	❑ 2	❑ 3	❑ 4	❑ 88
**8.15**	**The female condom provides women with another contraceptive choice.**	❑ 1	❑ 2	❑ 3	❑ 4	❑ 88
**8.16**	**The female condom provides women with another choice to protect themselves against both HIV and other sexually transmitted diseases.**	❑ 1	❑ 2	❑ 3	❑ 4	❑ 88
**8.17**	**Sex doesn't feel as good when you use a female condom.**	❑ 1	❑ 2	❑ 3	❑ 4	❑ 88
**8.18**	**Female condoms make it hard for a woman to have an orgasm (cum).**	❑ 1	❑ 2	❑ 3	❑ 4	❑ 88
**8.19**	**Female condoms make it hard for a man to have an orgasm (cum).**	❑ 1	❑ 2	❑ 3	❑ 4	❑ 88
**8.20**	**Female condoms take all the fun out of sex.**	❑ 1	❑ 2	❑ 3	❑ 4	❑ 88
**8.21**	**You don't like putting the female condom inside yourself.**	❑ 1	❑ 2	❑ 3	❑ 4	❑ 88
**8.22**	**You don't like having to touch yourself to put the female condom in.**	❑ 1	❑ 2	❑ 3	❑ 4	❑ 88
**8.23**	**You don't like having to use your finger to put the female condom in.**	❑ 1	❑ 2	❑ 3	❑ 4	❑ 88
**8.24**	**If a woman wants to use a female condom, her partner might think she was having sex with someone else.**	❑ 1	❑ 2	❑ 3	❑ 4	❑ 88

9.0 FEMALE CONDOM SELF-EFFICACY

We know that for many women situations exist when it is very difficult to insist on female condom use. I am going to read you a list of different situations. We would like to learn which situations are more difficult for you. Please think about each one and tell me how confident you are that you could do them if you decided it was important to you. For each statement, tell me if you are 'very sure', 'somewhat sure', 'somewhat unsure', or 'very unsure'. *THESE QUESTIONS ARE TO BE ASKED OF ALL PARTICPANTS, EVEN IF THEY DO NOT CURRENTLY HAVE A PARTNER.* **OPTION CARD 1**

**How confident are you that…**		**Very sure**	**Somewhat sure**	**Somewhat unsure**	**Very unsure**
**9.1**	**You can discuss female condom use with any sexual partner you might have?**	❑ 1	❑ 2	❑ 3	❑ 4
**9.2**	**You can insist on female condom use if a partner does not want to use one?**	❑ 1	❑ 2	❑ 3	❑ 4

**9.3**	**You can insist on female condom use with a person who gets angry when you suggest it?**	❑ 1	❑ 2	❑ 3	❑ 4
**9.4**	**You can start to insist on female condom use with a long-time partner with whom you haven't used condoms in the past?**	❑ 1	❑ 2	❑ 3	❑ 4
**9.5**	**You can insist on female condom use regularly, even when you're under the influence of alcohol or drugs?**	❑ 1	❑ 2	❑ 3	❑ 4
**9.6**	**You can convince a new partner to use a female condom?**	❑ 1	❑ 2	❑ 3	❑ 4
**9.7**	**You can say 'no' to sex if your partner won't use a female condom?**	❑ 1	❑ 2	❑ 3	❑ 4
**9.8**	**You can use a female condom correctly?**	❑ 1	❑ 2	❑ 3	❑ 4

10.0 MALE CONDOMS
Now I am going to ask you some questions about male condoms.

10.1 *MARK IF USED MALE CONDOMS IN LAST 2 MONTHS*. **If you have used male condoms in the last TWO months, from where do you and your partner/s usually get them?**

Place	Self	Partner/s
This facility/site	❑	❑
Another facility/site<br>*SPECIFY TYPE NGO/WORKPLACE*	❑<br>________________________	❑<br>________________________
Pharmacy	❑	❑
Shop	❑	❑
Friend	❑	❑
Don't know where partner gets them	❑	❑
Other, *SPECIFY*	❑<br>________________________	❑<br>________________________

**10.2**	**Did you get any male condoms during your visit today?**<br><br>*MARK ALL THAT CLIENT MENTIONS*	❑ 1= Yes, male condoms from provider<br>❑ 2= Yes, male condoms from dispenser<br>❑ 3= No, I did not get any male condoms	
**10.3**	**How effective do you think the male condom is at preventing HIV and other STIs?**<br><br>**Would you say 'extremely effective', 'somewhat effective', 'somewhat ineffective', or 'extremely ineffective'?**	❑ 1= Extremely ineffective<br>❑ 2= Somewhat ineffective<br>❑ 3= Somewhat effective<br>❑ 4= Extremely effective<br>❑ 88= Don't know<br><br>*SHOW OPTION CARD 2*	

**11.0 MALE CONDOM SELF-EFFICACY**<br>**We know that for many women situations exist when it is very difficult for them to insist on male condom use. I am going to read you a list of different situations. We would like to learn which situations are more difficult for you. Please think about each one and tell me how confident you are that you could do them if you decided it was important to you. For each statement, tell me if you are 'very sure', 'somewhat sure', 'somewhat unsure', or 'very unsure'.** *OPTION CARD 1*					
**How confident are you that…**		**Very sure**	**Somewhat sure**	**Somewhat unsure**	**Very unsure**
**11.1**	**You can discuss male condom use with any sexual partner you might have?**	❑ 1	❑ 2	❑ 3	❑ 4
**11.2**	**You can insist on male condom use if a partner does not want to use one?**	❑ 1	❑ 2	❑ 3	❑ 4
**11.3**	**You can insist on male condom use with a person who gets angry when you suggest it?**	❑ 1	❑ 2	❑ 3	❑ 4
**11.4**	**You can start to insist on male condom use with a long-time partner with whom you haven't used condoms in the past?**	❑ 1	❑ 2	❑ 3	❑ 4
**11.5**	**You can insist on male condom use regularly, even when you're under the influence of alcohol or drugs?**	❑ 1	❑ 2	❑ 3	❑ 4
**11.6**	**You can convince a new partner to use a male condom?**	❑ 1	❑ 2	❑ 3	❑ 4
**11.7**	**You can say 'no' to sex if your partner won't use a male condom?**	❑ 1	❑ 2	❑ 3	❑ 4
**11.8**	**You and your partner can use a male condom correctly?**	❑ 1	❑ 2	❑ 3	❑ 4

**12.0 HIV RISK AND TESTING**<br><br>**Now I am going to ask you some questions about HIV. Some of the questions may be sensitive but remember, our conversation will be confidential and you may refuse to answer any questions.**			
**12.1**	**How would you rate yourself in terms of risk of becoming infected with HIV?**	❑ 1= I will definitely not get infected with HIV<br>❑ 2= I probably won't get infected<br>❑ 3= I am probably going to get infected<br>❑ 4= I am definitely going to get infected with HIV<br>❑ 5= I am HIV positive already	
**12.2**	**Have you ever had an HIV test, today or any other time?**	❑ 0 = No<br>❑ 1 =Yes<br>❑ 99 = Refused	IF 1, GO TO Q12.3<br><br>IF 2, GO TO Q12.5
**12.3**	I **would like to ask you if you would be prepared to share the results of your HIV test with me. This information will be kept confidential (I will not tell anybody else) and there is no name on this questionnaire. If you do not want to tell me, that's no problem. We can skip this question.**	❑ 1= Positive<br>❑ 2= Negative<br>❑ 3= Did not get results<br>❑ 4= Do not wish to disclose	IF 2,3,OR 4, GO TO Q12.5<br><br>IF 1, GO TO Q12.4

**12.4**	**If HIV-positive, are you currently taking ARVs?**	❑ 0= No<br>❑ 1= Yes<br>❑ 99= Refused	GO TO Q 12.5
**12.5**	**Do you know your partner's HIV status?**	❑ 0 = No<br>❑ 1 =Yes<br>❑ 88 = Don't know<br>❑ 99 = Refused	
**12.6**	I **would like to ask you if you would be prepared to share the results of your partner's (s)' HIV status with me. This information will be kept confidential and there is no name on this questionnaire. If you do not want to tell me, that's no problem. We can skip this question.**	❑ 1= Positive<br>❑ 2= Negative<br>❑ 3= Did not get results<br>❑ 4= Did not wish to disclose<br>❑ 88 = Don't know	IF 2,3,OR 4, GO TO 13.0<br><br>IF 1, GO TO Q12.7
**12.7**	**If partner is positive, is he on ARVs?**	❑ 0= No<br>❑ 1= Yes<br>❑ 88 = Don't know<br>❑ 99 = Refused	

**13.0 SEXUALLY TRANSMITTED INFECTIONS**<br>**Now I am going to ask you some questions about sexually transmitted infections. Some of the questions may be sensitive but remember, our conversation will be confidential and you may refuse to answer any questions.**			
**13.1**	**In the last year, have you had or has a health care provider told you that you had a sexually transmitted infection? – an infection that can be transmitted through sexual contact**	❑ 0 = No<br>❑ 1 =Yes<br>❑ 88 = Don't know/can't remember<br>❑ 99 = Refused	IF 0,<br>GO TO<br>END
**13.2**	*IF ANSWERED 'YES' TO Q13.1*<br>**What symptoms did you have?**	❑ 1= Abnormal vaginal discharge<br>❑ 2= Ulcer or warts<br>❑ 3= Genital discomfort/itching/burning/rash<br>❑ 4= Thrush (Candida)<br>❑ 5= Cystitis (bladder infection)<br>❑ 6= Lower abdominal pain<br>❑ 7= Pain during sexual intercourse<br>❑ 77= Other, *SPECIFY*<br>______________________________	
**13.3**	**What did the provider offer you?** *PROMPT*		
**a.**	**Pap smear, or test for cervical cancer screening**	❑ 0= No<br>❑ 1= Yes<br>❑ 88= Can't remember	
**b.**	**Male condoms**	❑ 0= No<br>❑ 1= Yes<br>❑ 88= Can't remember	
**c.**	**Demonstration on how to use a male condom**	❑ 0= No<br>❑ 1= Yes<br>❑ 88= Can't remember	

**d.**	**Female condoms**	❑ 0= No<br>❑ 1= Yes<br>❑ 88= Can't remember	
**e.**	**Demonstration on how to use a female condom**	❑ 0= No<br>❑ 1= Yes<br>❑ 88= Can't remember	
**f.**	**HIV counselling and testing**	❑ 0= No<br>❑ 1= Yes<br>❑ 88= Can't remember	
**g.**	**Medication to treat the STI**	❑ 0= No<br>❑ 1= Yes<br>❑ 88= Can't remember	
**h.**	**Counselling on how to take medication for the STI**	❑ 0= No<br>❑ 1= Yes<br>❑ 88= Can't remember	
**i.**	**Instructions on how to notify your sexual partner(s) about their need to be assessed for possible STI**	❑ 0= No<br>❑ 1= Yes<br>❑ 88= Can't remember	

That's the very last question. Thank you so much for taking the time to participate in this interview. Do you have any questions now that we are finished?

IF PARTICIPANT REPORTED ANY TYPE OF ABUSE, ASK PARTICIPANT IF SHE WOULD LIKE A REFERRAL, AND IF SO, REFER TO PROVIDER/ GIVE HER THE LIST OF AGENCIES PROVIDING DOMESTIC VIOLENCE SERVICES.

IF PARTICIPANT REPORTED SHE OR HER PARTNER IS HIV-POSITIVE (Q12.3 OR Q12.6), ASK PARTICIPANT WHETHER SHE OR HER PARTNER NEED A REFERRAL FOR HIV CARE AND REFER TO PROVIDER /PROVIDE LIST OF HEALTH CENTRES.
