# Supplementary material for: Lessons from the evaluation of the South African National Female Condom Programme
Source: PLoS One. 2020 Aug 13;15(8):e0236984. doi: 10.1371/journal.pone.0236984 (PMC7425948; doi:10.1371/journal.pone.0236984)
Supplement: S5 File — (PDF) [file pone.0236984.s005.pdf]

Evaluation of the National South African Female Condom Programme: Investigating Factors Associated with Uptake and Sustained Use Facility/Site Telephonic/In-Depth Assessment

1.1	Interviewer Code	[___|___]	1.6	Interviewee Name	
1.2	Facility/Site ID	[___|___]___|___]___]	1.7	Interviewee Designation	
1.3	Facility/Site Phone Number	[__|__|__]<br>[__|__|__|__|__|__|__|__]	1.8	Location:	❑ 1= Rural<br>❑ 2= Urban<br>❑ 3= Peri-urban
1.4	Facility/Site Name		1.9	DoH sites only-Facility:	❑ 1= PHC<br>❑ 2= CHC<br>❑ 3= Hospital, Mobile
1.5	Facility/Site Organisation	❑ 1= DoH<br>❑ 2= Other, SPECIFY	1.10	Length of FC distribution	❑ 1= >5 years<br>❑ 2= 2-5 years<br>❑ 3= <2 years

Visit	Date	Commencement Time	Completion Time	Outcome	Comments
1.11	___ /___ /___<br>dd/mm/yy	_____:_____<br>H H : M M	_____:_____<br>H H : M M		
1.12	___ /___ /___<br>dd/mm/yy	_____:_____<br>H H : M M	_____:_____<br>H H : M M		

Outcome Codes:

❑ **1**= Interview complete
❑ **2**= Person not available
❑ **3**= Interview partially complete
❑ **4**= Appointment made for another Interview
❑ **5**= Refused to participate in the study

Thank you for being willing to complete this telephonic/in-depth facility/site assessment today.

Telephonic Script

Thank you for agreeing to take part in this telephone survey. There is more detail in the information flyer your site received, but if you would like me to go through this information again, I am happy to do so [*GO THROUGH SCRIPT OR INFO SHEET IF PERSON RESPONDING HAS NOT SEEN THE INFORMATION LEAFLET*]. This telephonic interview will take about 30 minutes of your time. Is this a convenient time?

In-depth Script

Your facility/site completed a telephonic survey on the (*MENTION DATE*) and with (*PERSON*) [*IF SOMEONE DIFFERENT FROM THE PERSON THE ASSESSMENT IS BEING CONDUCTED WITH TODAY WITH A MatCH RESEARCH TEAM MEMBER*]. This interview will confirm some of the key information that was collected in that telephone call and some additional details about your female and male condom distribution. Is this a convenient time to do the interview?

INTERVIEW QUESTIONS

*PUT AN **X** IN THE APPROPRIATE BOX(ES) FOR ALL QUESTIONS*

**CURRENT DISTRIBUTION OF CONDOMS**		
**1.**	**Have you ever distributed female condoms?**<br>❑ 1= Yes<br>❑ 2= No	**IF YES,GO TO Q2**<br>**IF NO, GO TO Q6**
**2.**	**In which year did you first distribute FCs?**<br>*IF NOT KNOWN AT THE FACILITY, WRITE 'NOT KNOWN'*<br>**Year**______________<br>❑ 88= Don't know	**GO TO Q3**
**3.**	**Do you currently distribute FCs (Any distribution in last month)?**<br>❑ 1= Yes<br>❑ 2= No	**IF NO, GO TO Q4**<br>**IF YES, GO TO Q7**
**4.**	**When did you stop distributing FCs?**<br>Year___________<br>Month_____________	**GO TO Q5**
**5.**	**a. Why have you stopped distributing FCs?**<br>*PUT AN X IN THE BOX FOR ALL RESPONSES THAT APPLY*<br>❑ 1= Stock-out<br>❑ 2= FCs in stock, but no staff trained<br>❑ 3= No demand-- have not reordered<br>❑ 4= FCs in stock, but other reasons for non-distribution<br>*GIVE REASONS*,<br>____<br>____<br>❑ 77 = Other,<br>*PLEASE EXPLAIN*<br>____<br>____<br>**b. Please give more details on responses given above (e.g. length of stock-out)**<br>____<br>____<br>____	**GO TO Q6**
**6.**	**What actions, if any, are being taken to start/ restart FC distribution?**<br>*PUT AN X IN THE BOX FOR ALL RESPONSES THAT APPLY*<br>❑ 1= FCs on order waiting for delivery<br>❑ 2= Clinic requested staff training<br>❑ 3= No action, we are not ordering/reordering<br>(*SPECIFY WHY NOT*)______________________________<br>❑ 77= Other,<br>*PLEASE EXPLAIN*<br>____<br>____	**IF NEVER DISTRIBUTED FCS, GO TO Q27**
**7.**	**Have you had any stock expire in the last year?**<br>❑ 1 = Yes<br>❑ 2 = No<br>❑ 88 = Not sure/don't remember	**GO TO Q8**

IN THIS SECTION (Q8-Q18), WE ARE GOING TO ASK YOU QUESTIONS ABOUT THE STEPS INVOLVED IN RECORDING AND DOCUMENTING FC DISTRIBUTION.		
**8.**	**What is recorded when a client is given FCs? For example, if a client is given 5 FCs, what and where is this recorded?**<br>*IF ONLY RECORDED IN CERTAIN CIRCUMSTANCES SUCH AS WITHIN SITE DISTRIBUTION, EXPLAIN BELOW AND COMPLETE BOTH Q9 AND Q10*<br>❑ 1= Number of FCs given noted in register/book<br>❑ 2= Who was given FC (female/male/age?)<br>❑ 3= Type of FC (e.g. FC2/Cupid, etc.)<br>❑ 4= No individual record kept<br>❑ 77= Other,<br>*PLEASE EXPLAIN*____	**IF 1 OR 2, GO TO Q9**<br>**IF 3, GO TO Q10**<br>**IF 4, COMPLETE Q10**
**9.**	**Are these individual records written in registers/books collated/added up to get total clients and total FCs distributed over time?**<br>*PUT AN X IN BOX FOR ALL RESPONSES THAT APPLY*<br>❑ 1= Yes – total FCs distributed are counted/totalled and reported from these registers<br>❑ 2= Yes – total clients taking FCs is counted/totalled from these registers<br>❑ 3= No – these registers/records are not used to report total FC distribution	**IF 1 OR 2, GO TO Q12**<br>**IF 3, GO TO Q11**
**10.**	**If no individual records kept, how do you report FC distribution?** (E.g. boxes used – how is this recorded?)/no report required/just request when stocks low or used up)<br>*PLEASE EXPLAIN*____	**GO TO Q12**
**11.**	**If you record FC distribution but do not use your registers/record books to report FC distribution outside your site, how do you report total FC distribution?** (E.g. boxes used– how is this recorded?)/no report required/ just request when stocks low or used up)<br>*PLEASE EXPLAIN*____	**GO TO Q12**
**12.**	**To whom do you report the facility/site FC distribution data (including client numbers if available)?** *PUT AN **X** IN THE APPRROPRIATE BOX(ES)*	

	Reported to whom (organisation/level)	Frequency of reporting, e.g. monthly	FCs reported in what units?	Are client numbers reported?
**a.**			❑ 1= Numbers<br>❑ 2= Boxes	❑ 1= Yes<br>❑ 2= No
**b.**			❑ 1= Numbers<br>❑ 2= Boxes	❑ 1= Yes<br>❑ 2= No
**c.**			❑ 1= Numbers<br>❑ 2= Boxes	❑ 1= Yes<br>❑ 2= No

13. Who do you order your condoms from when you need stock? *PUT AN **X** IN THE APPROPRIATE BOX(ES) FOR BOTH FEMALE AND MALE CONDOMS*

ALL GO TO Q14

Supply site name		Female condoms	Male condoms
a.		❑ 1= Yes<br>❑ 2= No	❑ 1= Yes<br>❑ 2= No
b.		❑ 1= Yes<br>❑ 2= No	❑ 1= Yes<br>❑ 2= No
c.		❑ 1= Yes<br>❑ 2= No	❑ 1= Yes<br>❑ 2= No

14. Is the reporting site the same as the ordering site? *PROBE FOR RESPONSES IN Q12 & Q13 FOR CONFIRMATION*

❑ 1 = Yes
❑ 2 = No
❑ 3 = Other, *PLEASE EXPLAIN*

____
____

15. Can you describe your ordering system for condoms? *PUT AN **X** IN THE APPROPRIATE BOX(ES)*

ALL GO TO Q16

Ordering system	Female condoms	Male condoms
**Paper-based (faxed or scanned)**	❑ 1= Yes<br>❑ 2= No	❑ 1= Yes<br>❑ 2= No
**Paper-based (posted, delivered or collected)**	❑ 1= Yes<br>❑ 2= No	❑ 1= Yes<br>❑ 2= No
**Email direct request/on-line**	❑ 1= Yes<br>❑ 2= No	❑ 1= Yes<br>❑ 2= N0
**Telephone**	❑ 1= Yes<br>❑ 2= No	❑ 1= Yes<br>❑ 2= No
**Other, *SPECIFY***	______________<br>________	____________<br>_______

16. In the last 6 months, <u>on average</u> how long does it take to get condom stock from date of your order to it arriving at your site? If you collect your FCs, how long after your order can you go and collect them? *WRITE DOWN TO NEAREST WEEK. IF LESS THAN A WEEK, WRITE <1*

	Supply site name	Delivery mode	Female condoms	Male condoms
**1.**		❑ 1= Delivered<br>❑ 2= Collected	________ weeks	________ weeks
**2.**		❑ 1= Delivered<br>❑ 2= Collected	________ weeks	________ weeks
**3.**		❑ 1= Delivered<br>❑ 2= Collected	________ weeks	________ weeks

17. When did you last order FCs? How many FCs did you order?

a. Month/year of last order ___________/_____________
Month Year
b. How many FCs did your site order? ____________

ALL GO TO Q18

18. **When do you usually order female condoms? Do you order when stock falls below a certain number, last box, or do you order at a regular time, e.g. monthly/quarterly?**

❑ 1= Order when we are down to our last box
❑ 2= Order when we have below a certain number on the Stock card (specify)__________
❑ 3= Order at specific times (monthly/quarterly) Specify_________
❑ 77= Other *SPECIFY*_________________

ALL GO TO Q19

19. **a. How many FCs did you distribute in (3-month time period**).

IF IT IS NOT POSSIBLE TO CALCULATE DUE TO REPORTING ISSUES (E.G. ONLY REPORTING WHEN BOX IS EMPTY), ESTIMATE HOW MANY BOXES ARE USED ON AVERAGE PER MONTH.

IF ANY STOCK-OUT IN THESE MONTHS, WRITE "YES". IF FCS WERE AVAILABLE BUT NO FCS WERE DISTRIBUTED, WRITE "O".

**Month**	**FCs distributed N [No FCs distributed =0]**			**Any FC stock-out?**		**Includes FC distribution to other sites?**		**Discrepancy between DHIS, Tel. and in-depth?**
	**DHIS** ______	**Tel** ______	**SV**	**Tel**	**SV**	**Tel**	**SV**	
**Feb 2014**	______	______	_______	❑ 1=Yes ❑ 2=No	❑ 1=Yes ❑ 2=No	❑ 1=Yes ❑ 2=No	❑ 1=Yes ❑ 2=No	❑ 1=Yes ❑ 2=No
**March 2014**	______	______	_______	❑ 1=Yes ❑ 2=No	❑ 1=Yes ❑ 2=No	❑ 1=Yes ❑ 2=No	❑ 1=Yes ❑ 2=No	❑ 1=Yes ❑ 2=No
**April 2014**	______	______	_______	❑ 1=Yes ❑ 2=No	❑ 1=Yes ❑ 2=No	❑ 1=Yes ❑ 2=No	❑ 1=Yes ❑ 2=No	❑ 1=Yes ❑ 2=No

b. What is your source of information for the numbers you provided?

❑ 1= Clinic statistics
❑ 2= Estimate based on Stock card
*PLEASE EXPLAIN*____
____

c. If there is a discrepancy, probe where it may have arisen from? (During site visit)

____
____

Questions 20 and 21 are for facilities/sites that have a current FC stock-out (Q5) or mention a stock-out in any of the 3 months in Q19. *IF NO STOCK-OUT RECORDED, GO TO Q22*

20. **During the FC stock-out you reported in (Confirm month given by participant in Q5/Q19) or are currently experiencing, why do you think the stock-out occurred?**

❑ 1= We ordered on time, but FCs were late or did not come
❑ 2= We ordered late (after stock ran out or just before)
❑ 77= Other, *SPECIFY*________________________

21. **During this current/past stock-out, have you [made/did you make] any attempts to get a supply of FCs from another site?**

❑ 1= Yes
❑ 2= No

Questions 22 -23 are about FCs you may give to other organisations/clinics. If you conduct no sub-distribution, GO TO Q24		
**22.**	**Has your facility/site provided FCs from your own stock to other sites e.g. NGOs, clinics, other community organisations in the last 3 months?** *CHECK AGAINST Q19*<br>❑ 1= Yes<br>❑ 2= No	**IF NO, GO TO Q24**<br><br>**IF YES, GO TO Q23**

23. Can you tell us to whom you have given FCs?

THIS QUESTION SHOULD INDICATE SUB- DISTRIBUTION ***(SD)*** *TO OTHER SITES/ORGANISATIONS WHO CONDUCT THEIR OWN DISTRIBUTION, OR STAFF FROM THIS SITE WHO DISTRIBUTE FCs IN ANOTHER SITE/ORGANISATION.INCLUDE 3 MOST RECENT SITES.*

	SD site 1	SD site 2	SD site 3
**Name/location**			
**Organisation type- e.g. NGO, clinic.**			
**Number of branches/sub-sites(n)**			
**Number of FCs given in last 3 months (total)**			
**FC's collected from & delivered by**	❑ 1= Collected from this facility<br>❑ 2= Delivered by this facility	❑ 1= Collected from this facility<br>❑ 2= Delivered by this facility	❑ 1= Collected from this facility<br>❑ 2= Delivered by this facility
**FC distribution**	❑ 1= FCs distributed by SD site<br>❑ 2= FCs distributed by site staff<br>❑ 3= FCs distributed by SD and site staff	❑ 1= FCs distributed by SD site<br>❑ 2= FCs distributed by site staff<br>❑ 3= FCs distributed by SD and site staff	❑ 1= FCs distributed by SD site<br>❑ 2= FCs distributed by site staff<br>❑ 3= FCs distributed by SD and site staff

24. Which brands of FCs have you ever distributed?(FC,Cupid and pleasuremore)

PUT AN ***X*** *IN THE BOX FOR ALL RESPONSES THAT APPLY. SOME CLINICS MAY STILL HAVE FC1 STOCKS. IF PARTICIPANT ANSWERS 'NOT SURE', PROBE FOR PACKAGING TYPE*

❑ 1= FC2
❑ 2= FC1
❑ 3= Cupid
❑ 4= Pleasuremore
❑ 5= Woman's Condom
❑ 6= Not sure if FC1 or FC2
❑ 88= Not sure
❑ 77= Other *SPECIFY*_____________________________________

25. **Where are your condoms stored BEFORE they are put in dispensers/ consulting rooms, etc?**
NEED TO KNOW STORAGE SITE N.B. STORAGE AND DISPENSING SITE Q26 MAY BE THE SAME

Storage Location	FCs	MCs	Adequate storage	Expired
**Store room/dispensary**	❑ 1= Yes<br>❑ 2= No	❑ 1= Yes<br>❑ 2= No	❑ 1= Yes<br>❑ 2= No	❑ 1= Yes<br>❑ 2= No
**Office/consulting room**	❑ 1= Yes<br>❑ 2= No	❑ 1= Yes<br>❑ 2= No	❑ 1= Yes<br>❑ 2= No	❑ 1= Yes<br>❑ 2= No
**Waiting area (including clerks desk)**	❑ 1= Yes<br>❑ 2= No	❑ 1= Yes<br>❑ 2= No	❑ 1= Yes<br>❑ 2= No	❑ 1= Yes<br>❑ 2= No
**Other, *SPECIFY***	________ ________ ________	________ ________ ________	________ ________ ___	________ ________

If not adequate storage (site visit only) check storage issue and please give details in observation section

❑ 1= Cartons/boxes in direct sunlight
❑ 2= Cartons/boxes stored on ground/against walls in storerooms
❑ 3= Cartons/boxes stored in damp/wet conditions
❑ 4= Other, *SPECIFY* ____
____

26. **Where do you dispense FCs?** *NEED TO KNOW HOW MANY OF EACH TYPE OF DISTRIBUTION POINT AT SITE AND IF THEY ARE PRIVATE, E.G. CLIENTS NOT OBSERVED TAKING CONDOMS*

Location	Total N of type of distribution point	Type e.g. box, dispenser can, basket	Stocked/ Empty	Total N in private location	Resupply If 2: Probe and write what Fast track system
**Office/ consulting room**			❑1=Stocked<br>❑2= Empty		❑1=Queue<br>❑2=Fast track/no queue
**Waiting area (including clerks desk)**			❑1=Stocked<br>❑2=Empty		❑1=Queue<br>❑2=Fast track/no queue
**Toilets male only**			❑1=Stocked<br>❑2= Empty		❑1=Queue<br>❑2=Fast track/no queue
**Toilets female only**			❑1=Stocked<br>❑2=Empty		❑1=Queue<br>❑2=Fast track/no queue
**Toilets unisex**			❑1=Stocked<br>❑2=Empty		❑1=Queue<br>❑2=Fast track/no queue
**Corridors**			❑1=Stocked<br>❑2=Empty		❑1=Queue<br>❑2=Fast track/no queue
**Outside building (at gate/on external wall)**			❑1=Stocked<br>❑2=Empty		❑1=Queue<br>❑2=Fast track/no queue
**Other *SPECIFY***	________ ________ ____	________ ________	________ ________	________ ________ ____	________ ________

27. **a. How many MCs did you distribute in (3-month time period)?**

IF IT IS NOT POSSIBLE TO CALCULATE DUE TO REPORTING ISSUES SUCH AS ONLY REPORTING WHEN BOX IS EMPTY, ESTIMATE HOW MANY BOXES ARE USED ON AVERAGE PER MONTH AND THEIR CONTENT NUMBER.

IF ANY STOCK-OUT IN THESE MONTHS, WRITE "YES" IF MCS WERE AVAILABLE BUT IF NONE DISTRIBUTED, WRITE "O".

GO TO Q28 THIS WILL BE CHECKED IN REGISTERS

**Month**	**Number distributed**			**Any MC stock-out?**	
	**DHIS**	**Tel**	**SV**	**Tel**	**SV**
**Feb 2014**				❑ 1= Yes<br>❑ 2= No	❑ 1= Yes<br>❑ 2= No
**March 2014**				❑ 1= Yes<br>❑ 2= No	❑ 1= Yes<br>❑ 2= No
**April 2014**				❑ 1= Yes<br>❑ 2= No	❑ 1= Yes<br>❑ 2= No

b. What is your source of information for the numbers you provided?
❑ 1= Clinic statistics
❑ 2= District/province data
❑ 3= Estimate based on ? *PLEASE EXPLAIN*______
____

28. **Where are your male condom dispensers/cans/baskets located?**
NEED TO KNOW HOW MANY AND IF THEY ARE PRIVATE, E.G CLIENTS NOT OBSERVED TAKING CONDOMS. READ OUT OPTIONS IF NOT DISTRIBUTED AT THAT LOCATION WRITE""0""

**Location**	**Total N of distribution point**	**Type e.g. box, dispenser, can, basket**	**Stocked/Empty**	**Total N in private location**
**Office/consulting room**			❑ 1= Stocked<br>❑ 2= Empty	
**Waiting area/ clerks desk**			❑ 1= Stocked<br>❑ 2= Empty	
**Toilets male only**			❑ 1= Stocked<br>❑ 2= Empty	
**Toilets female only**			❑ 1= Stocked<br>❑ 2= Empty	
**Toilets unisex**			❑ 1= Stocked<br>❑ 2= Empty	
**Corridors**			❑ 1= Stocked<br>❑ 2= Empty	
**Outside site/ gate/ wall**			❑ 1= Stocked<br>❑ 2= Empty	
**Other *SPECIFY***______	__________<br>__________<br>__	______________<br>________	__________________<br>__________	_________<br>_______

29.	**Can you tell me what condom-specific (FC/MC) IEC materials you have in the clinic and also any non-specific IEC materials (only referring to condoms not male or female)?**	**GO TO Q30. THIS WILL BE CHECKED IN IN-DEPTH VISIT**

TICK '1'FOR YES AND '2' FOR NO;

ADD NUMBER OF OFFICES/CONSULTING ROOMS (WHERE CLIENTS ARE SEEN) IN COLUMN 3.

BE SURE TO CHECK THAT NUMBER OF ROOMS DOES NOT EXCEED TOTAL REPORTED IN Q26. READ OUT EACH TYPE OF IEC.

**IEC**	**Waiting area**		**Offices/consulting rooms (give proportion, e.g. 2/4)**		**Other places (name)**	
	Tel	SV	Tel	SV	Tel	SV
**FC leaflets**	❑ 1= Yes<br>❑ 2= No	❑ 1= Yes<br>❑ 2= No	__/__	__/__		
**FC posters**	❑ 1= Yes<br>❑ 2= No	❑ 1= Yes<br>❑ 2= No	__/__	__/__		
**FC demonstration models***	❑ 1= Yes<br>❑ 2= No	❑ 1= Yes<br>❑ 2= No	__/__	__/__		
**Dual protection FC and another method**	❑ 1= Yes<br>❑ 2= No	❑ 1= Yes<br>❑ 2= No	__/__	__/__		
**MC leaflets**	❑ 1= Yes<br>❑ 2= No	❑ 1= Yes<br>❑ 2= No	__/__	__/__		
**MC posters**	❑ 1= Yes<br>❑ 2= No	❑ 1= Yes<br>❑ 2= No	__/__	__/__		
**MC demonstration models/dildos**	❑ 1= Yes<br>❑ 2= No	❑ 1= Yes<br>❑ 2= No	__/__	__/__		
**Condom non-specific leaflets**	❑ 1= Yes<br>❑ 2= No	❑ 1= Yes<br>❑ 2= No	__/__	__/__		
**condom non-specific posters**	❑ 1= Yes<br>❑ 2= No	❑ 1= Yes<br>❑ 2= No	__/__	__/__		
**Dual protection MC and another method**	❑ 1= Yes<br>❑ 2= No	❑ 1= Yes<br>❑ 2= No	__/__	__/__		

***For FC demonstration models, please explain what type you have available.**
CHECKED DURING OBSERVATION WALKABOUT

____

____

30.	**Has your site/facility conducted any health talks to clients that include male or female condom promotion/information in the last 3 months**? *IF 'DON'T KNOW', ASK IF ANY OTHER STAFF MEMBER WOULD KNOW. ONLY MARK 'DON'T KNOW' IF THERE IS NO INFORMATION AVAILABLE*<br>❑ 1= No, all condom counselling only given on one-to-one basis in last 3 months<br>❑ 2= Yes<br>❑ 88= Don't know/unsure	**IF NO, GO TO Q32**<br>**IF YES, GO TO Q31**
31.	**Can you tell me how often the talks were conducted, what was included, and where they were conducted? Ask to review Health talk records**	

	Male condoms	Female condoms
**How many talks have you done (N)?**		
**Where were the talks done?**	❑ 1= Waiting area<br>❑ 2= Other, *SPECIFY* ______________ ______________	❑ 1= Waiting area<br>❑ 2= Other, *SPECIFY* ______________ ______________
**What was included?**	❑ 1= Verbal information<br>❑ 2= Demonstration<br>❑ 3= Other, *SPECIFY* ______________ ______________	❑ 1= Verbal information<br>❑ 2= Demonstration<br>❑ 3= Other, *SPECIFY* ______________ ______________

32.	**Can you tell me about staff trained in Female Condom provision and distribution in your site?**	

	Nurse (all categories)	Counsellors/ CCGs	Other (clerk, guard, GA)
**Total number of staff**			
**No. of staff routinely providing FC counselling and distribution**			
**No. of these staff ever trained in FCs**			
**Others trained (not routinely providing the service)**			

OBSERVATION CHECKLIST

Please observe and fill in the details in the following table:

**33. Registers**<br>*ASK TO SEE THE REGISTERS AS PER Qs 8-9. IS THE INFORMATION THE SAME AS REPORTED IN THESE QUESTIONS? IF NOT, EXPLAIN BELOW*
**34. Condom Storage**<br>*ANY ADDITIONAL COMMENTS/OBSERVATIONS ON STORAGE OF THAT REPORTED IN Q25*
**35. Condom Dispensers**<br>*ANY ADDITIONAL COMMENTS/ OBSERVATIONS ON THE DISPENSERS. IS THERE A FAST TRACK QUEUE FOR FCs AS REPORTED IN Q26?*

**36. Condom IEC**<br>*ANY ADDITIONAL COMMENTS/OBSERVATIONS ON THE IEC OR DEMONSTRATION MODELS* OR TALKS
**37. General comments**
